# Systematic analysis of transcription start sites in avian development

**DOI:** 10.1371/journal.pbio.2002887

**Published:** 2017-09-05

**Authors:** Marina Lizio, Ruslan Deviatiiarov, Hiroki Nagai, Laura Galan, Erik Arner, Masayoshi Itoh, Timo Lassmann, Takeya Kasukawa, Akira Hasegawa, Marian A. Ros, Yoshihide Hayashizaki, Piero Carninci, Alistair R. R. Forrest, Hideya Kawaji, Oleg Gusev, Guojun Sheng

**Affiliations:** 1 Division of Genomic Technologies, RIKEN Center for Life Science Technologies (CLST), Yokohama, Japan; 2 RIKEN Omics Science Center (OSC), Yokohama, Japan; 3 Institute of Fundamental Medicine and Biology, Kazan Federal University, Kazan, Russia; 4 International Research Center for Medical Sciences (IRCMS), Kumamoto University, Kumamoto, Japan; 5 RIKEN Center for Developmental Biology, Kobe, Japan; 6 Instituto de Biomedicina y Biotecnología de Cantabria, IBBTEC (CSIC-Universidad de Cantabria-SODERCAN), Santander, Spain; 7 RIKEN Preventive Medicine and Diagnosis Innovation Program, Yokohama, Japan; 8 Harry Perkins Institute of Medical Research, QEII Medical Centre and Centre for Medical Research, the University of Western Australia, Nedlands, Western Australia, Australia; 9 RIKEN Innovation Center, Wako, Japan; The Francis Crick Institute, United Kingdom of Great Britain and Northern Ireland

## Abstract

Cap Analysis of Gene Expression (CAGE) in combination with single-molecule sequencing technology allows precision mapping of transcription start sites (TSSs) and genome-wide capture of promoter activities in differentiated and steady state cell populations. Much less is known about whether TSS profiling can characterize diverse and non-steady state cell populations, such as the approximately 400 transitory and heterogeneous cell types that arise during ontogeny of vertebrate animals. To gain such insight, we used the chick model and performed CAGE-based TSS analysis on embryonic samples covering the full 3-week developmental period. In total, 31,863 robust TSS peaks (>1 tag per million [TPM]) were mapped to the latest chicken genome assembly, of which 34% to 46% were active in any given developmental stage. ZENBU, a web-based, open-source platform, was used for interactive data exploration. TSSs of genes critical for lineage differentiation could be precisely mapped and their activities tracked throughout development, suggesting that non-steady state and heterogeneous cell populations are amenable to CAGE-based transcriptional analysis. Our study also uncovered a large set of extremely stable housekeeping TSSs and many novel stage-specific ones. We furthermore demonstrated that TSS mapping could expedite motif-based promoter analysis for regulatory modules associated with stage-specific and housekeeping genes. Finally, using *Brachyury* as an example, we provide evidence that precise TSS mapping in combination with Clustered Regularly Interspaced Short Palindromic Repeat (CRISPR)-on technology enables us, for the first time, to efficiently target endogenous avian genes for transcriptional activation. Taken together, our results represent the first report of genome-wide TSS mapping in birds and the first systematic developmental TSS analysis in any amniote species (birds and mammals). By facilitating promoter-based molecular analysis and genetic manipulation, our work also underscores the value of avian models in unravelling the complex regulatory mechanism of cell lineage specification during amniote development.

## Author summary

Early development cannot be studied in humans. Analysis of embryogenesis using avian models, which are phylogenetically closely related to mammals, can help us understand the complex regulatory mechanism of cell lineage specification during human development. We monitored the 3 weeks of chicken embryonic development from newly-laid egg to the point of hatching via CAGE expression profiling and revealed the first avian genome-wide set of genuine transcription start sites (TSSs) critical for differentiation and maturation from pregastrulation to hatching. By analyzing stage-specific expression profiles, we have identified enriched transcription factors responsible for lineage commitment and their corresponding regulatory modules. In addition, we reported a set of stable housekeeping genes more suitable for cross-sample normalization and calibration. Finally, we demonstrated the utility of a CAGE-based TSS dataset in developmental studies. Using the *Brachyury* gene as an example, we showed that CRISPR/Cas9-based genome-editing tools can be efficiently employed to target and transcriptionally activate the promoters of virtually any endogenous genes, enabled by the knowledge of their precise TSS locations. Our data, made available for easy exploration through the open ZENBU platform, represent an invaluable resource to study early development in amniotes.

## Introduction

Single-molecule sequencing technology in combination with Cap Analysis of Gene Expression (CAGE) allows mapping and quantification of transcription start sites (TSSs) at single base pair resolution [1,2]. Application of this technology to a large collection of human and mouse samples, covering almost all known cellular steady states, generated a comprehensive overview of mammalian promoter usage and revealed a complex architecture of cell type–specific and ubiquitously expressed gene promoters and their transcription factor occupancies [3–5]. Integration of such high-throughput TSS profiling data from vertebrate, invertebrate, and fungal model organisms is giving us a fresh look into eukaryotic promoter structure and evolution [6].

Understanding developmental ontogeny of steady states in the adult requires time-course analysis of undifferentiated or differentiating embryonic and fetal tissues. Doing so is ethically prohibited in the human and technically demanding in the mouse for early-stage embryos. Among the 975 human samples profiled in Functional Annotation of Mammalian Genome (FANTOM) 5 for CAGE-based TSSs, human embryo was not included; of the mouse developmental samples similarly profiled (approximately a quarter of the total 399), all were from embryos older than E11 (halfway through their development) [3]. Such limitations can be circumvented by using nonmammalian vertebrate models, in which early embryonic samples are relatively easy to acquire. In a recent study using the zebrafish model, for example, an early developmental process called maternal-to-zygotic transition was analyzed through CAGE-based TSS profiling, and 2 fundamentally different transcription initiation codes were uncovered to regulate maternal and zygotic gene transcription [7,8].

In this work, we chose the chick as an amniote model for the exploration of ontogenetic evolution of TSS dynamics and generated genome-wide TSS profiles using embryonic samples covering the entire period of chick development, from egg-laying to hatching [9]. Both birds and mammals are amniotic vertebrates. Their development is evolutionarily conserved and highly stereotypic. Among all nonmammalian model organisms currently in use, the chick is the best experimental system for human developmental studies [10,11]. About 60% of the 22,000 human protein-coding genes have 1-to-1 orthologs in the chick [12–14]. Developmental programs leading to the formation of the 3 principal germ layers and their subsequent lineage specification are highly similar between these 2 species [10]. Importantly, the most critical period of vertebrate lineage diversification, gastrulation and early postgastrulation development, taking place after implantation and a difficult time for tissue isolation in most mammalian species, is readily accessible in the chick model. To achieve our aim, we collected chicken embryos from 16 developmental stages and carried out a comprehensive developmental CAGE-based TSS analysis of the chicken genome.

## Results and discussion

### Sample description and sequencing

**Fig 1:**
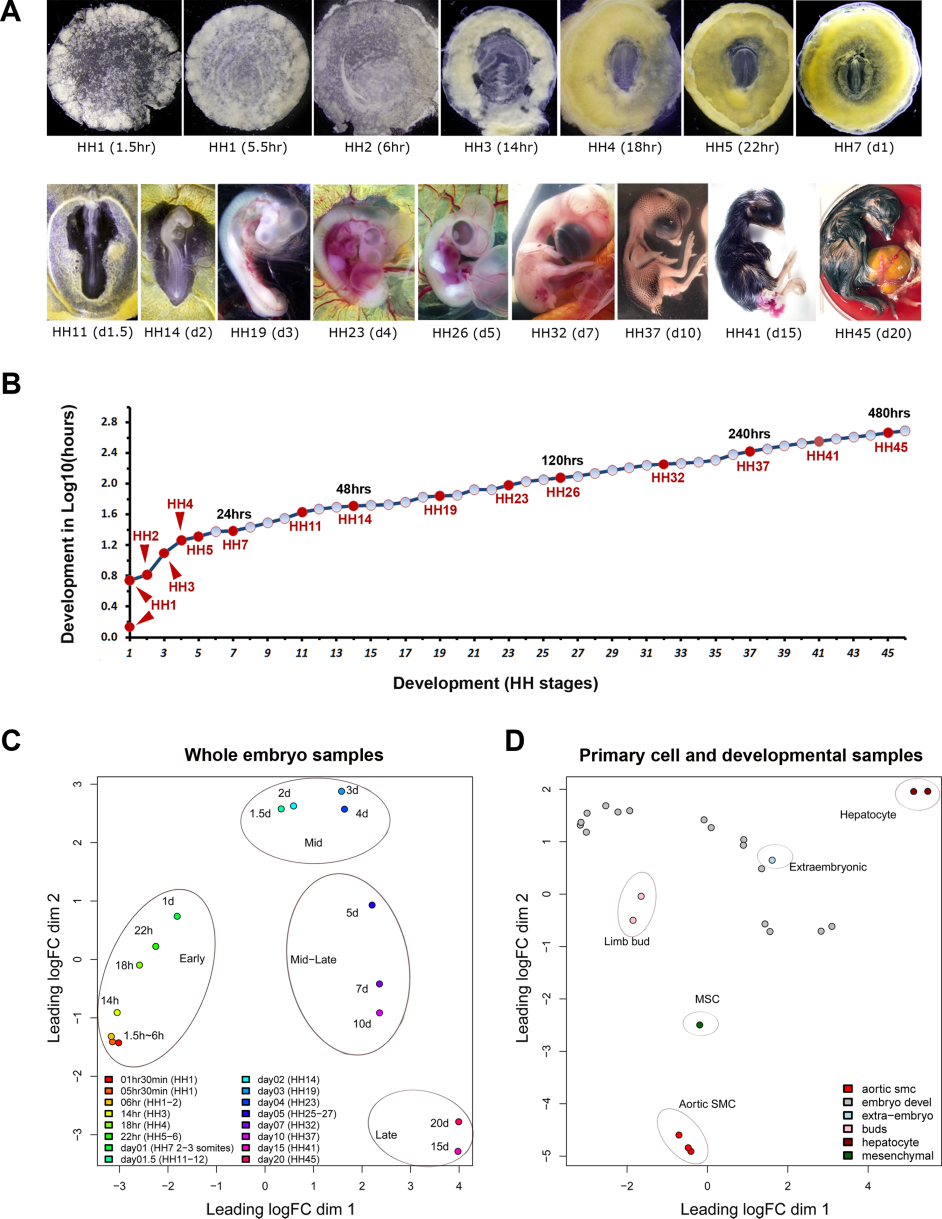
Developmental stages selected for Cap Analysis of Gene Expression (CAGE) study. (A) Representative images of chicken embryos used for RNA preparation. (B) Temporal distribution of these selected stages (red dots) during chicken embryogenesis. X-axis: 46 Hamburger and Hamilton (HH) stages represented in numerical order. Y-axis: log scale of incubation time needed to reach these stages (based on reported estimates), e.g., Y value of 2.0 = 100 hours. Numerical values for this plot can be found in S1 Data. (C) Principal coordinates analysis (PCoA) of all 16 developmental samples. Four groups, representing early, mid, mid-late, and late developmental phases, can be recognized. (D) PCoA of all samples. Primary cells are clearly grouped away from each other and from developmental samples. Raw data for PCoA can be accessed at http://fantom.gsc.riken.jp/5/suppl/Lizio_et_al_2017/.

Chicken samples from 16 developmental stages (Fig 1A and 1B; S1 Table), covering the entire prehatching developmental period from Hamburger and Hamilton (HH) stage 1 (HH1) to HH45 [9], were collected. At HH1, most of the cells in the embryo are pluripotent [15–17]; at HH45, most have reached a steady state after terminal lineage differentiation [18–20]. Ten additional samples of both embryonic and primary cell origins (2 limb bud, 2 extraembryonic, 3 aortic smooth muscle, 2 hepatocyte, and 1 bone marrow-derived mesenchymal stem cell [MSC]) were included for comparison (S1 Table). RNAs isolated from these samples were used for CAGE library construction and sequencing following established protocols [3] (Materials and methods). CAGE reads were mapped to galGal5, the latest chicken genome assembly (Genbank ID: GCA_000002315.3; NCBI link https://www.ncbi.nlm.nih.gov/assembly/GCA_000002315.3/). The median read depth for all chicken samples was 2.7 million, comparable to that of 4 million for human samples [3].

### CAGE-based TSS mapping

Peaks of TSS activity were identified across the genome using a decomposition-based peak identification (DPI) method [3]. In total, 142,924 peaks were identified with a permissive threshold of at least 1 TSS having 3 tag counts in 1 of the samples. Among them, 31,863 peaks remained after filtering with the robust threshold that requires 1 TSS with more than 10 tag counts in 1 sample (equivalent of 1 normalized tag per million [TPM]). Similar to the ratios seen in CAGE-based TSS analyses with human and mouse samples [3], 60% (19,172) of all robust chicken TSSs could be mapped to annotated TSSs of coding or noncoding gene models within 500 base pairs (based on chicken RefSeq, Ensembl, and EST databases for coding genes and chicken NONCODE [21], ALDB-lincRNA [22], and other lincRNA databases [23] for noncoding genes) (S1 Fig). The remaining 40% (12,644) of unannotated TSSs represented likely alternative promoters and noncoding RNA genes, which are yet to be characterized. About 31%–46% of all robust TSSs in any given sample (median 10,557 TSSs; 33% of total TSSs) had peak values greater than 3 TPM (S1 Table). Following the general practice in CAGE analysis, their corresponding genes were considered to have an abundance of roughly 1 transcript per cell and were categorized as expressed [3,24].

### Principal coordinates analysis

Because development is a continuous process, promoter activity profiles from different stages are causally linked. To assess sample-stage relationships based on their genome-wide TSS landscape, principal coordinates analysis (PCoA, a multiple-dimensional scaling approach) was performed. PCoA of all developmental samples revealed close correlation between TSS-based sample distance and embryonic stage–based developmental progression (Fig 1C). Samples from the first day of chicken embryogenesis, covering from pregastrulation to early somite stages (roughly corresponding to the second and third weeks of human development) were tightly clustered, suggesting that these stages possess shared TSS features. Among the remaining samples, groups representing mid development (D1.5–D4; from cardiac to hepatic differentiation), mid-late development (D5–D10; from definitive hematopoiesis to chondrogenesis), and late development (D15 and D20; bone and feather formation and terminal differentiation of most other cell lineages) could be readily distinguished (Fig 1C). When all samples were compared together, primary cells formed separate clusters (Fig 1D), reflecting transcriptional homogeneity in purified cell types, regardless of whether they represented the differentiated or multipotent lineages. The extraembryonic and limb bud samples, representing subpopulations of developmental tissues, were seen to group more closely to the whole embryos than to primary cells (Fig 1D).

### Chicken-ZENBU: An interactive platform for genome-wide TSS visualization

**Fig 2:**
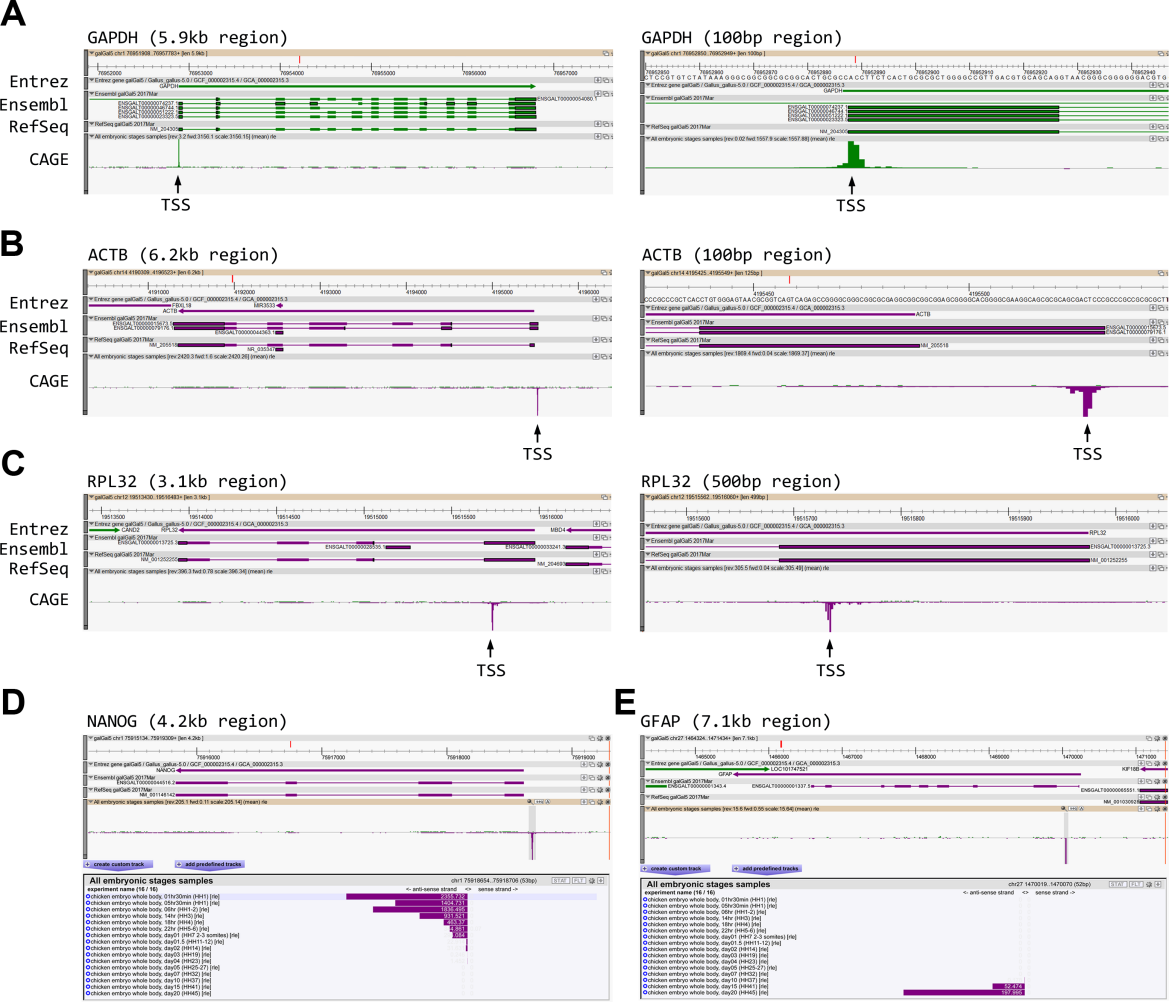
Chicken-ZENBU views of transcription start site (TSS) peaks and expression levels. (A) A robust single TSS peak (arrow) is correctly mapped to the known Ensembl and RefSeq 5′ end of *GAPDH*. Right panel: zoomed-in view of left panel. (B) Ambiguity in *ACTB* gene annotation can be resolved using Cap Analysis of Gene Expression (CAGE). (C) The TSS representative of *RPL32* gene does not confirm either available annotation, suggesting the incorrectness of both gene models. (D) Bar graph of expression values shows *NANOG* pluripotency gene present at early stages, then down-regulated at later stages. (E) Late stage–specific expression of *GFAP* gene (Hamburger and Hamilton stage 41 [HH41] and HH45). Samples in the bar graphs are sorted by developmental stage.

ZENBU genome browser was developed for the visualization of human CAGE data [25,26]. We adopted this platform and created a chicken-based ZENBU configuration (hereafter called Chicken-ZENBU; example shown in S2 Fig) for interactive and web-based exploration of chicken CAGE data (accessible at http://fantom.gsc.riken.jp/zenbu/gLyphs/#config=b1zZI1gUFZ6mHX6-4Gvxr). Briefly, Chicken-ZENBU allowed visual representation of TSS positions and peak values on galGal5, the latest version of chicken genome assembly. It was also fully integrated with the latest chicken genome annotation resources, including Ensembl, RefSeq, EST, NONCODE, ALDB-lincRNA, and highly conserved elements (HCEs) from 48 representative avian species [27,28]. Genes and TSSs could be searched and visualized at desirable genomic resolutions, from single nucleotide to chromosome levels. This allowed intuitive assessment of TSS consistency across all available sample data points. For example, inspection of TSS mapping results for the housekeeping gene *GAPDH* (Fig 2A; full view in S2 Fig) revealed in all samples a robust and single peak at the 5′ end of RefSeq transcript NM_204305 and Ensembl transcript *ENSGALT00000023323*.*5*, covering a 5 base pair–wide region and with the peak center mapped with single base pair precision to Chr1:76,952,888 (Fig 2A, right). This lends very strong support to the accuracy of both RefSeq and Ensembl gene models for *GAPDH*. In many cases, however, RefSeq and Ensembl gene models diverge on TSS prediction, and our CAGE-based TSS mapping could resolve such discrepancies (e.g., for beta actin gene *ACTB*, Fig 2B). When neither of 2 gene models matched our CAGE-based TSS mapping (e.g., for ribosomal protein gene *RPL32*, Fig 2C), future revision in annotation is recommended.

### Stage- and cell type–specific TSSs

In addition to genome-wide precision mapping of chicken TSSs, our data allowed us to track changes in TSS positions and peak values throughout chicken development. With regard to genomic position, half of mapped annotated genes had a single associated TSS peak in all samples (S2 Table), indicating that these genes are transcribed from a stably positioned TSS regardless of developmental stage or cellular origin. The other half of the genes exhibited 2 or more TSS peaks (S2 Table), likely reflecting alternative promoter utilization. This ratio is comparable to alternative promoter utilization reported for the human [3] and *Drosophila* [29] genomes.

**Fig 3:**
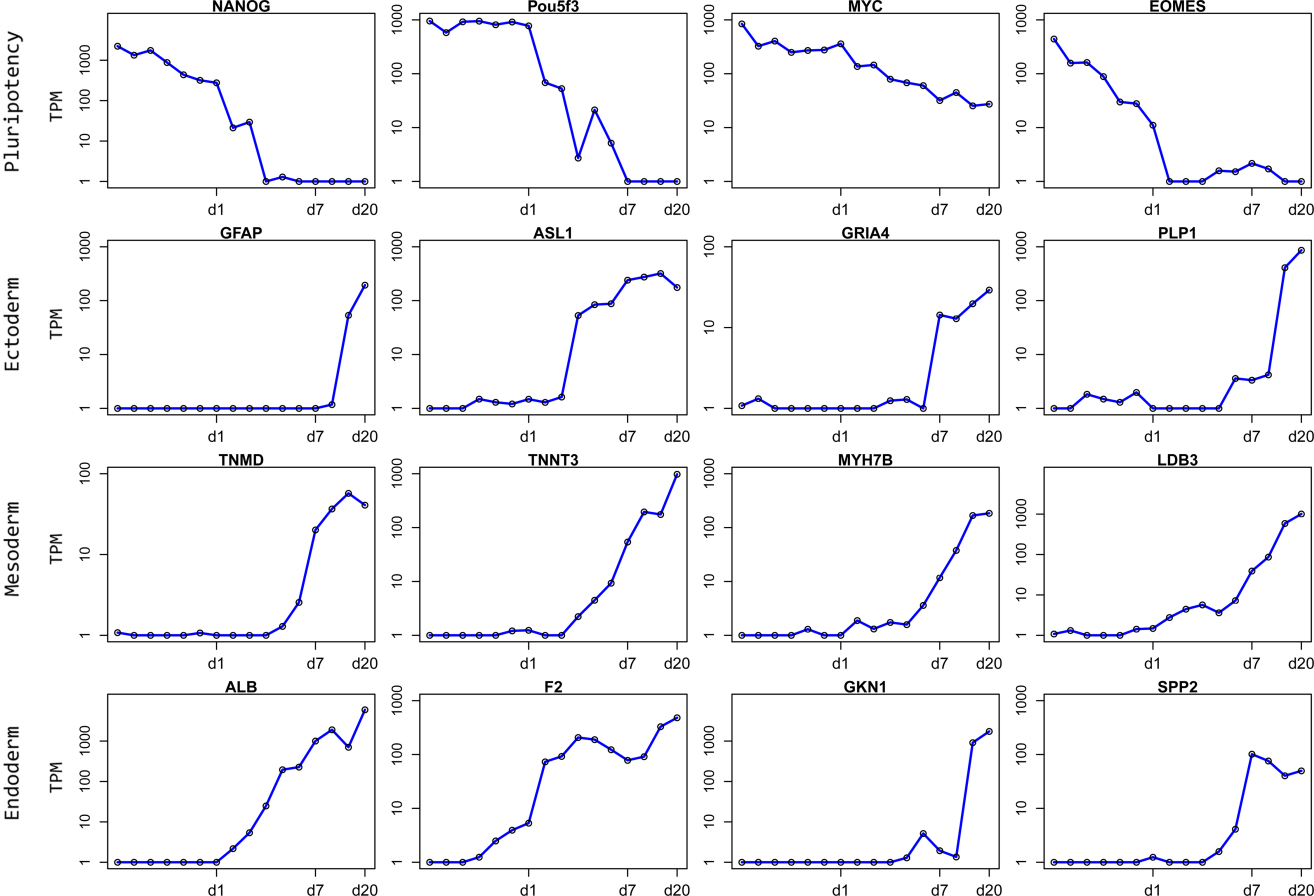
Expression profiles of stage- and cell type–specific transcription start sites (TSSs). Cap Analysis of Gene Expression (CAGE) TSSs associated with pluripotency and germ layer–specific genes show distinct expression patterns during development. Pluripotency-related genes (*NANOG*, *POU5F3*, *MYC*, *EOMES*) show early stage–specific expression. Ectoderm-, mesoderm-, and endoderm-related genes show opposite expression patterns, being activated at later stages of development. X-axis represents developmental stages; y-axis represents tag per million (TPM) expression values on a logarithmic scale. Numerical values for this plot can be found in S1 Data.

TSS peak values, on the other hand, were stable only for a small percentage of all reported TSSs (see the housekeeping gene section below). For most TSSs, their peak values exhibited prominent variation among developmental samples. Such variation could be detected in our analysis with high sensitivity, capturing CAGE TSS peak values spanning 5 orders of magnitude. This allowed us to make quantitative assessment of promoter activities in heterogeneous cell populations in the embryo. For example, loss of epiblast pluripotency during the first day of embryonic development was clearly revealed by tracking expression changes of the TSS peak of the *NANOG* gene (Chicken-ZENBU views in Fig 2D and S3A Fig; quantification in Fig 3). Similar changes were observed for many other pluripotency-associated genes (e.g., *POU5F3*, *MYC*, and *EOMES*) (Fig 3). Likewise, following the developmental progression, lineage-specific differentiation in each of the 3 principal germ layers (the ectoderm, mesoderm, and endoderm) could be readily assessed. For instance, a single TSS peak was detected in the *GFAP* locus (Chicken-ZENBU views in Fig 2E and S3B Fig; quantification in Fig 3), and its rapid rise in activity in the second half of embryogenesis marked the differentiation of astrocytes, a derivative of the ectoderm germ layer. Furthermore, TSS profiles for many other ectoderm-derived, lineage-specific markers, such as *ASL1* (lens), *GRIA4* (neurons), and *PLP1* (oligodendrocytes), and for mesoderm- and endoderm-derived lineage-specific markers (e.g., *TNMD* [tendon], *TNNT3* [fast-twitch muscle], *MYH7B* [slow-twitch muscle], and *LDB3* [striated muscle] for the mesoderm and *ALB*, *F2*, and *SPP2* [liver] and *GKN1* [stomach] for the endoderm) could also be precisely tracked throughout development (Fig 3). Interestingly, in a subset of genes with multiple associated TSS peaks, each peak exhibited unique developmental profiles, likely reflecting alternative promoter activities under stage-specific transcriptional regulation. For example, the *CDYL* gene, encoding an uncharacterized chromodomain protein, had 2 robust TSS peaks (S4A Fig). One of them (TSS1) was expressed ubiquitously (S4B and S4C Fig), whereas the other (TSS2) showed high levels of expression only during early development (S4B and S4C Fig).

To evaluate stage- and cell type–specific TSSs systematically, we applied a promoter activity cutoff of 10 TPM and asked whether any TSSs had over 10-fold enrichment over the cross-sample mean in any particular sample group. In total, 2,296 TSSs were found to be highly enriched in primary cells (1,175 in hepatocytes, 449 in aortic smooth muscle cells [SMCs], and 672 in MSCs) (S3 Table) and 608 TSSs in developmental samples (172 in early stage samples, 118 in mid stage samples, 184 in mid-late stage samples, 493 in late stage samples, 292 in extraembryonic samples, and 81 in limb samples) (S3 Table). It is worth noting that many of these stage- or cell type–specific promoter activities had been previously unreported or poorly characterized (examples shown in S5 Fig).

### A complete list of housekeeping TSSs and novel candidates for extremely stable TSSs

Many genes are involved in the maintenance of basic cellular machinery and are expressed ubiquitously in all cell types. Some of them are also highly expressed (e.g., genes encoding GAPDH, tubulin, actin, and ribosomal proteins). These genes are commonly used as loading/calibration controls in expression analysis. However, recent studies have shown that most of such housekeeping genes are expressed neither uniformly nor stably across different tissue/cell types [30]. In our study, out of the 31,863 TSS peaks identified in the chicken genome, 3,631 (11.4%) were active in all 26 samples (>3 TPM) and could be considered as ubiquitously expressed. Among them, 189 (0.59%) were highly expressed (>100 TPM) and 79 (0.25%) were extremely highly expressed in all samples (>300 TPM cutoff and >700 TPM cross-sample mean). Most of the commonly used housekeeping genes (e.g., *GAPDH* and *ACTB*) were in this last group (S4 Table).

**Fig 4:**
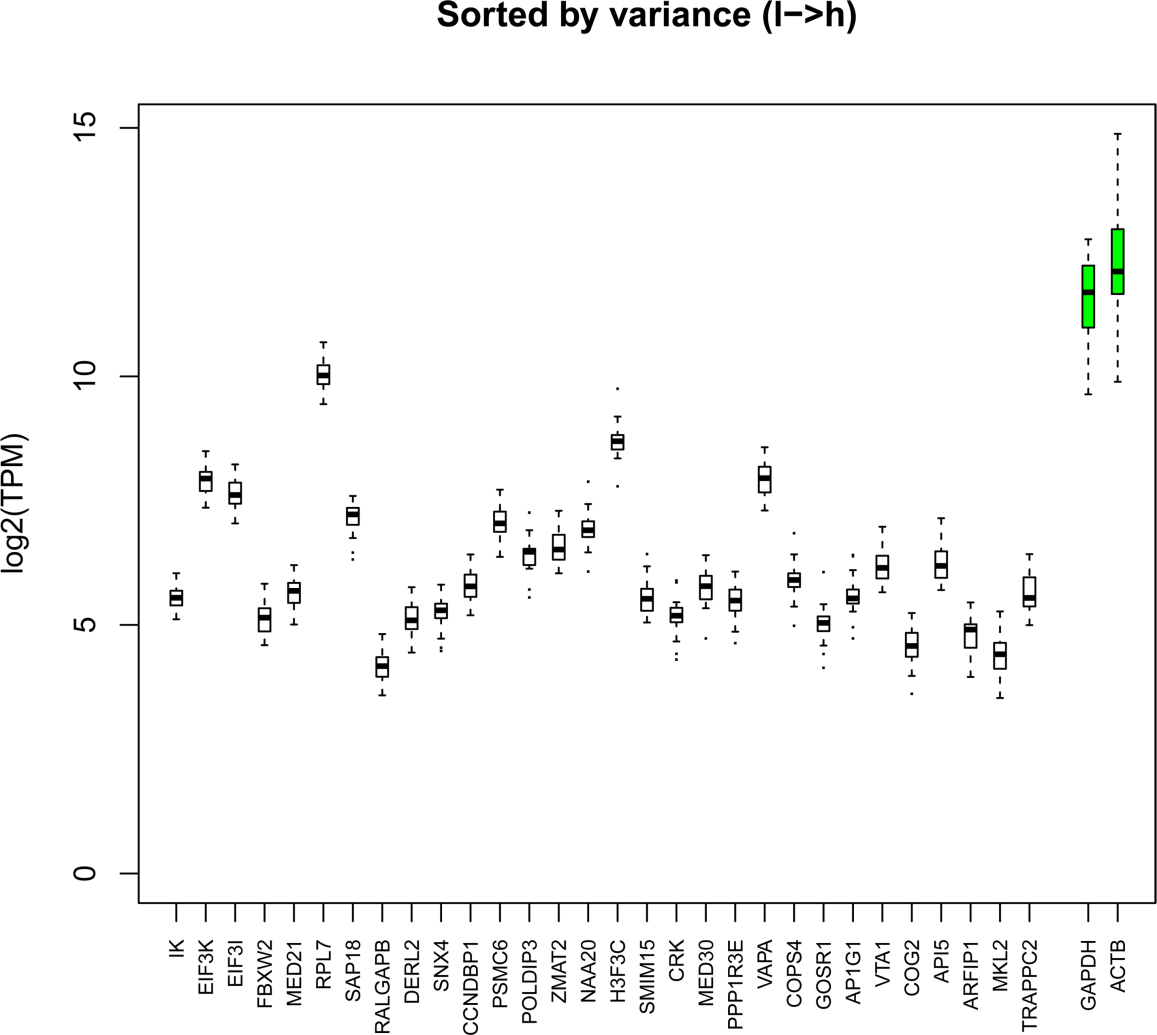
Chicken housekeeping genes. Expression in tags per million (TPM) across all 26 samples for the top 30 housekeeping genes with lowest variance is compared to expression of well-known housekeeping genes *GAPDH* and *ACTB*. Genes are listed by variance (low to high). Numerical values for this plot can be found in S1 Data.

Despite their ubiquitous and high levels of expression, however, very significant cross-sample variations were noted for these genes, an observation in support of Eisenberg and Levanon’s general assessment on housekeeping genes [30]. They applied alternative criteria (ubiquitous expression, low tissue variance, and no exceptional expression) and found 3,804 human genes as bona fide housekeeping genes. Using a similar approach (>10 TPM, σ(log_2_TPM)<1), we identified 1,254 chicken housekeeping genes (1,399 TSSs) (S5 Table), among which 787 (63%) were shared between the human and the chicken (S6 Table). Gene ontology (GO) analysis of all 1,254 chicken housekeeping genes indicated an exclusive functional association with basic cellular machinery, including translation, transcription, subcellular organization, metabolism, and protein trafficking (S7 Table). The vast majority of those housekeeping genes were expressed much more stably than *GAPDH* (ranked 1,077) and *ACTB* (not considered as a housekeeping gene according to this criterion) (S5 Table). Among the top 30 most stable genes (Fig 4), the top 10 in expression stability were *IK*, *EIF3K*, *EIF3I*, *FBXW2*, *MED21*, *RPL7*, *SAP18*, *RALGAPB*, *DERL2*, and *SNX4* (Fig 4); and the top 10 in expression levels were *RPL7*, *H3F3C*, *VAPA*, *EIF3K*, *EIF3I*, *NAA20*, *PSMC6*, *SAP18*, *ZMAT2*, and *POLDIP3* (S6 Fig). These genes are recommended as the bona fide housekeeping genes for expression normalization in avian studies.

### General features of chicken CAGE TSSs

Genome-wide TSS mapping enabled us to analyze general features of chicken promoters, such as CpG island overlap, GC content, length and shape, and their usage across stages and cell types. Based on bioinformatics analysis of the chicken genome, approximately 70,000 CpG islands in total were predicted, and 48% of them were associated with a gene [12,31]. We found that 13,701 out of all robust TSSs (43%) overlap a CpG island, a ratio higher than what was reported for human TSSs (33%) [3]. This is in agreement with an overall higher GC content in the chicken genome (chicken 52%; human 41%) [32]. We also analyzed the correlation of peak shape (as an index of precision in the choice of TSS) with other promoter features. Peak shape, whether broad or sharp, was assessed by “shape index” (SI) [33]. A sharp peak was defined as having SI > −1 and a broad peak as having SI < −1, with the highest possible SI being 2, representing 100% single-base position mapping of all tags. Broad peaks exhibited higher GC content (within a 100 bp region; ± 50 bp around the TSS representative position) (S7A Fig) and, consequently, showed higher presence of CG-rich motifs (S7B Fig bottom; position weight matrix visualization for aligned “sharp” and “broad” 100 bp promoter regions). Furthermore, they overlap CpG islands with higher frequency (S7C Fig), although the overall percentage of CpG-associated TSSs (among all expressed TSSs with >3 TPM) did not exhibit prominent variation among the samples (S7F Fig). Broad peaks were also associated with a greater bias towards non-tissue–specific genes (S7D and S7E Fig). Sharp peaks, on the other hand, were clearly associated with TATA box motif by position weight matrix visualization (S7B Fig top). These results agree with previous studies on the mammalian [3] and *Drosophila* [33] promoter architecture. We further investigated promoter usage by merging TSS peaks (>1 TPM) within 100 bp distance into super clusters (Materials and methods) [3]. In total, 21,632 super clusters were obtained; 14,422 (71%) of them had 1 TSS, and the remainder had 2 or more TSSs. A general shift to a broader super cluster size was seen with developmental samples (S7G Fig), suggesting that there is an overall trend to have more TSSs active as development proceeds.

### TSS mapping as a tool for systematic promoter and enhancer analysis

Systems-level studies of avian development are yielding unprecedented details on how transcriptional regulatory networks control lineage specification [34–38]. Such studies could be further aided by a genome-wide understanding of promoter and enhancer architecture, which has not yet been reported in the chick. Using data from 48 representative avian species, recent work on avian comparative genomics [27,28] showed that over 99% of approximately a quarter million Avian-Specific Highly Conserved Elements (ASHCEs; a subset of all HCEs) were located in noncoding regions of the genome, pointing to their potential roles in transcriptional regulation. Among the TSS peaks identified in this work, 110 (0.34%) directly overlapped the ASHCEs, and 2,660 (8.35%) directly overlapped the HCEs. Not surprisingly, GO analysis of the top 500 ASHCE-associated genes suggested a strong bias towards developmental functions [28]. To facilitate future analysis of ASHCEs in developmental regulation, we mapped all ASHCEs to galGal5 and integrated this information into Chicken-ZENBU (as HCE and ASHCE tracks; label 11 in S2 Fig). Examples of HCEs and ASHCEs in the *MSX1* and *MSX2* loci are shown in S8 Fig. Other vertebrate comparative genomics resources, such as the 0.6 million Conserved Non-Exonic Elements (CNEEs) identified through whole-genome alignment of a broad spectrum of vertebrate species [39], could, in the future, be similarly mapped and visualized on Chicken-ZENBU.

**Fig 5:**
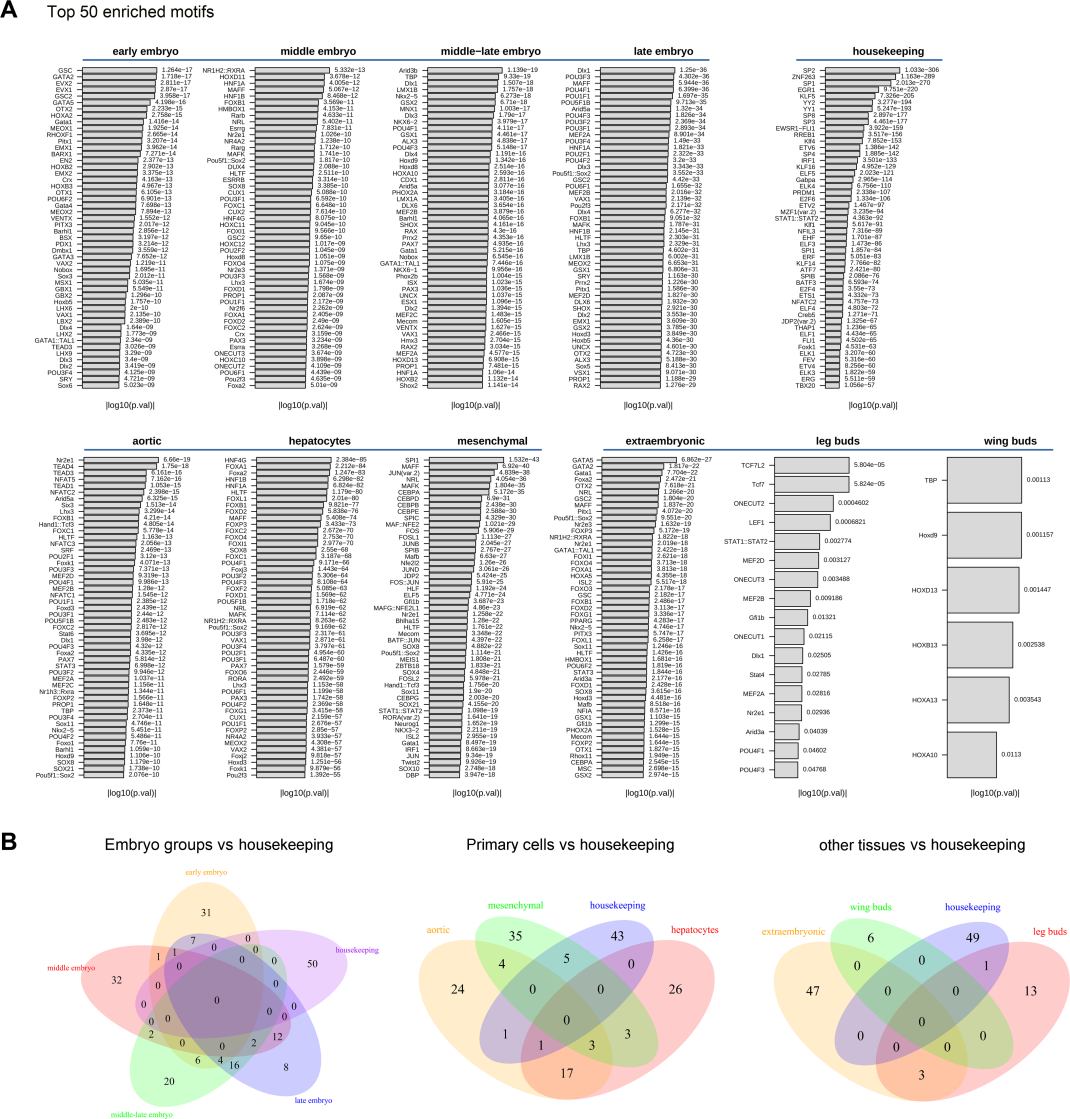
Shared and unique transcription factor binding site motifs in the vicinity of transcription start sites (TSSs) enriched in sample subgroups. (A) The top 50 enriched motifs in each subgroup are shown. Embryo group contains early, middle, middle-late, and late subgroups. Primary cells group contains mesenchymal stem cells (MSCs), aortic smooth muscle cells (SMCs), and hepatocytes subgroups. Other tissues group contains leg and wing buds and extraembryonic subgroups. (B) Venn diagram showing overlaps of those top 50 motifs for embryo, primary cells, and other tissues against housekeeping groups. The number of shared motifs between groups is low, suggesting stage- or cell type–specific regulatory programs.

In addition to allowing integration of conserved regulatory elements and TSS activities, our data also enabled systematic profiling for transcription factor binding sites (TFBSs) in any promoter(s) of interest. As a proof of principle, we asked whether lineage-specific binding motifs could be revealed through TFBS profiling of promoter sequences (arbitrarily defined as −300 to +100 base pair region) around the identified TSSs. TSSs enriched in each sample group (>10 TPM) (S3 Table) were profiled for TFBSs by using Analysis of Motif Enrichment (AME) motif-based sequence analysis tool (Meme-Suite.org, see Materials and methods) [40]. The top 50 motifs for enriched TSSs in each of the 10 sample categories and for housekeeping genes are shown in Fig 5A. All enriched motifs are shown in S8 Table. A small percentage of the top 50 motifs were shared among sample groups (0 for embryo groups, 3/50 for primary cells, and 0 for other tissues) or with the housekeeping gene group (0 for all) (Fig 5B, S9 Table). Most of the identified top 50 TFBS motifs were highly specific to individual sample category (31/50 for early embryo group, 32/50 for middle embryo group, 20/50 for mid-late embryo group, 8/50 for late embryo group, 24/50 for aortic SMCs, 35/50 for MSCs, 26/50 for hepatocytes, 47/50 for extraembryonic tissue, 6/6 for wing buds, and 13/17 for leg buds) (Fig 5B, S9 Table), suggesting that they were primarily engaged in lineage-specific transcriptional regulation. Similar results were obtained when the comparison was performed with top 100 TFBS motifs (S9 Fig). Moreover, when TFBS analysis was extended to all TSSs with either sharp or broad peaks (as described in the previous section), distinct groups of enriched motifs were seen for the sharp (TA-rich motifs, including TBP binding site TATAAA) and broad (CG-rich motifs) TSS peaks (S10 Fig). These data demonstrate that TSS mapping combined with TFBS profiling enables us to identify enriched regulatory motifs with potentially specific roles in developmental- and lineage-related transcriptional regulation.

### TSS mapping enables efficient activation of endogenous genes with CRISPR-on technology

**Fig 6:**
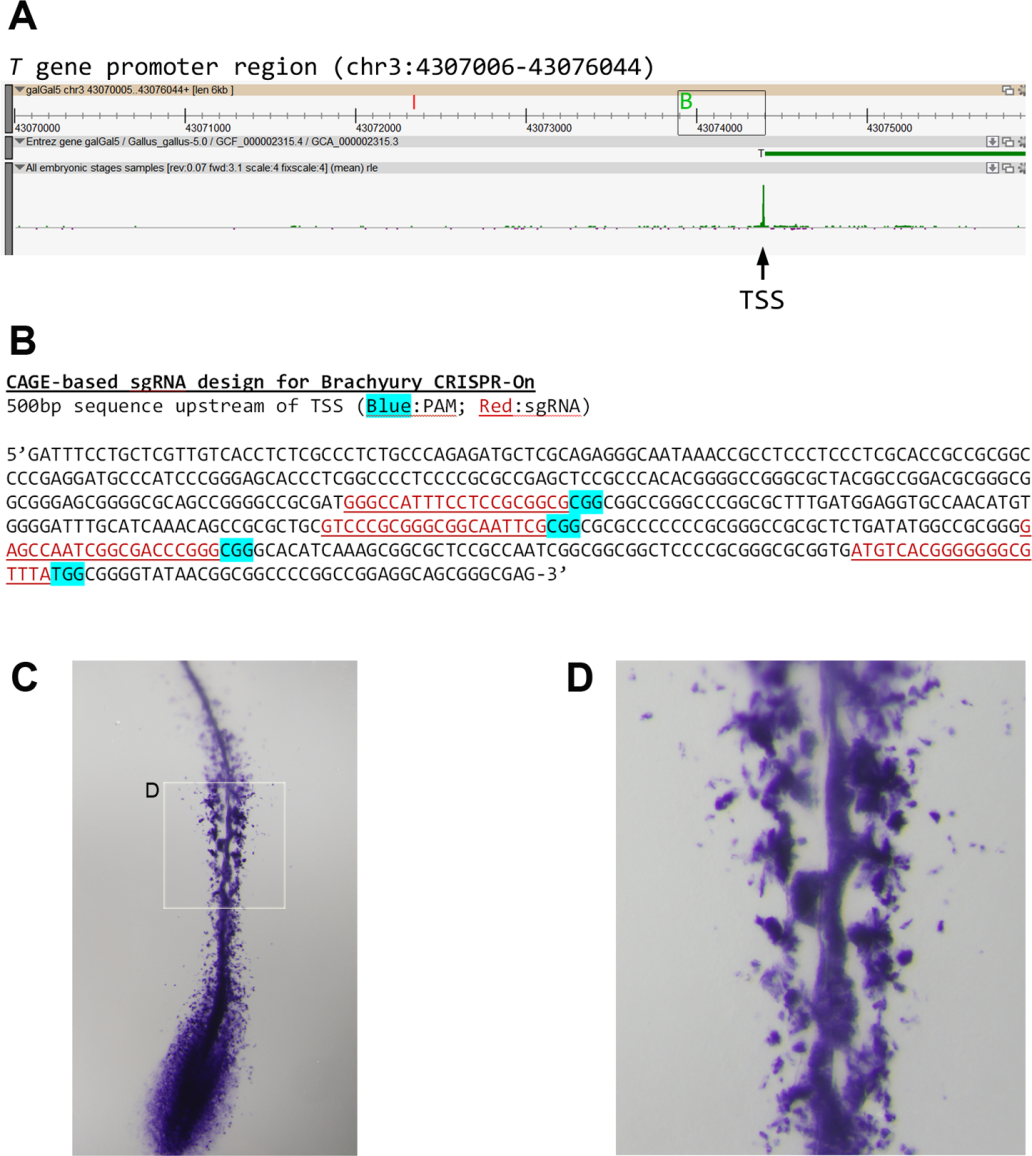
Induction of endogenous Brachyury expression mediated by Clustered Regularly Interspaced Short Palindromic Repeat (CRISPR)-on. (A) Chicken-ZENBU view of a 6-kb window on chromosome 3, showing the transcription start site (TSS) location for the *Brachyury* gene. Small rectangle represents the region shown in (B). (B) Sequences of 4 single guide RNAs (sgRNAs; red underline) located within 300 bp upstream of the *Brachyury* TSS. Blue: protospacer-adjacent motif (PAM). (C) Electroporated embryos were grown to Hamburger and Hamilton stage 10 (HH10), and sgRNA-expressing territories were assessed by co-electroporated GFP signal. Embryos were then processed for *Brachyury* in situ hybridization (shown here). (D) Magnified view of mid-region of the embryo, showing ectopic *Brachyury*+ cells in somites and neural tube.

Clustered Regularly Interspaced Short Palindromic Repeat (CRISPR)/Cas system is used by bacteria and archaea to recognize and cleave viral DNA sequences. As shown in mammalian models, this genome editing technology is also applicable to avian cells and embryos [41,42]. A modified Cas9 protein, with its nuclease activity dead and fused with 10 copies of VP16 transactivation domain, was able to recruit transcription initiation machinery to gene promoters guided by single guide RNA (sgRNA) [43]. This technique is termed “CRISPR-on” for CRISPR/dCas9-mediated gene activation. Previous studies showed that the highest efficiency of CRISPR-on was achieved by using multiple sgRNAs targeted to the first 200–300 base pairs upstream of TSS, whereas sgRNAs targeted to sequences further upstream had low efficiency and those targeted to sequences downstream of TSS had negative effect [43]. The CRISPR-on technique can potentially be used to activate any endogenous genes, many of which are ill-suited for conventional plasmid-based cloning and overexpression. Its success, however, relies heavily on the precise knowledge of TSSs of interest, and up to now, this technique has not been used in any avian or mammalian developmental study. To test whether our genome-wide TSS mapping could expedite CRISPR-on application in chick developmental analysis, we targeted the *Brachyury* (*T*) gene as a proof of principle for CRISPR-on–mediated activation. A single TSS peak was identified at the 5′ end of chicken *Brachyury* (Fig 6A), a gene marking mesoderm precursors and axial mesoderm cells at early developmental stages. The promoter region was analyzed for suitable sgRNA design (crispr.mit.edu) [44], and 4 sgRNA sequences located within 300 bp of the TSS were selected (Fig 6B). The expression construct “pAC154-dual-dCas9VP160-sgExpression” (addgene #48240) was used for sgRNA expression. Early gastrulation-stage embryos (HH2–HH3) were electroporated with combined 4 sgRNA-expressing constructs and were grown to HH10, followed by *Brachyury* in situ hybridization analysis. CRISPR-on–activated *Brachyury*+ signals were widely and ectopically seen in somitic and neural tube cells (Fig 6C and 6D) in addition to *Brachyury*’s normal expression domains in pre-ingressing mesoderm progenitors and notochord cells [45]. Several other genes tested were also robustly activated through CRISPR-on (S11 Fig and S12 Fig), suggesting that CRISPR-on technique (with the help of TSS mapping) can be efficiently applied to avian developmental research.

## Conclusions

TSSs were investigated in the past through nuclease protection, primer extension, and 5′ rapid amplification of cDNA end (RACE) assays. In the era of high-throughput sequencing, transcriptome profiling through RNA-seq massively expanded our ability to reconstruct transcript structure at the genome level. However, conventional RNA-seq techniques target random RNA fragments and are not best suited for accurate identification of TSSs [46]. CAGE technology, targeting and enriching 5′ capped RNA fragments, has emerged as the most widely adopted method for genome-wide TSS mapping, in comparison with other approaches such as dRNA-seq [47], TagRNA-seq [48], RNA annotation and mapping of promoters for analysis of gene expression (RAMPAGE) [29], and single-molecule real-time (SMRT) [49]. Applying the CAGE technology, we generated genome-wide TSS profiles for chicken embryos covering the entire developmental period from pregastrulation to hatching. Developmental TSSs were mapped to the latest chicken genome assembly and are open for interactive exploration on Chicken-ZENBU. Promoter activities reflecting complex lineage specification events, as well as housekeeping gene functions, were readily captured, yielding a rich resource for the analysis of ontogenetic evolution of steady state transcriptional activities in the adult. As the first genome-wide TSS mapping in birds and the first comprehensive developmental TSS analysis in amniotes, our data pave the way for the refinement of chicken genome annotation in combination with the latest improvement in genome assembly [14] and for systems-level analysis of promoters (for transcriptional regulation) and 5′ untranslated regions (for translational regulation) of developmentally important genes. As a proof of principle, we demonstrated that TSS profiling enabled us to find stage-dependent enriched TFBS motifs and to achieve high efficiency in CRISPR-on–mediated transcriptional activation of a mesoderm regulator. The latter is the first report of CRISPR-on technique in developmental studies, applicable in principle to any gene. CRISPR-on and the similar CRISPR-mediated transcriptional inhibition [50] and CRISPR-mediated epigenetic modification [51] techniques, all requiring precise TSS knowledge, have the potential to revolutionize developmental studies in model systems not amenable to traditional genetics-based analysis.

A recurring issue in high-throughput analysis is cellular heterogeneity regardless of sample source (e.g., a tumor or an embryo). This is being tackled in the field with the development of computational deconvolution algorithms on one hand [52–54] and of nanogram- and pictogram-scale RNA-seq platforms on the other, including CAGE-adapted nanoCAGE and C1 CAGE [55–57]. Unlike tumor tissues, which are inhomogeneous by nature and variable in their relative cellular composition, embryos are composed of highly stereotypic and reproducible cell populations, with their fates and contributions well mapped out. In our current analysis, early-stage embryos contain relatively few distinct cell populations, whereas late-stage embryos are composed of hundreds of steady state cell types. Early embryonic tissues are therefore ideally suited for testing and optimizing deconvolution algorithms. Sensitivity in CAGE peak identification also implies that minor cell populations can be readily detected and quantified in either simple or complex mixed populations. Chicken germ cells, for example, represent an extreme case in which only less than 0.1% of all embryonic cells are involved at any developmental stage. Yet TSSs representing germ cell–specific markers such as *DDX4* (*CVH*) could still be detected from stage HH1 to stage HH6, suggesting that most lineage specification events, even during late embryogenesis (when tissue complexity increases dramatically), are captured in our analysis. Taken together, we conclude that our CAGE-based developmental TSS profiling and proof-of-principle analyses will significantly advance avian and mammalian developmental studies. With future improvement in deconvolution, CAGE library-making and RNA-sequencing platforms, avian embryos offer a rare physiological scenario for systems-level study of ontogenetic evolution of cell lineage specification.

## Materials and methods

This work does not involve the use of human or mammalian tissues or embryos. All experiments involving the use of chicken embryos in this work were conducted in accordance with the animal experiment guidelines from the Kumamoto University and the RIKEN Center for Developmental Biology.

### Sample collection, embryo culture, electroporation and in situ hybridization

Fertilized hens’ eggs were purchased from a local farm (Shiroyama Farm, Kanagawa, Japan). Eggs were incubated at 38.5°C to desired stages. For HH1 to HH26 samples (1.5-hour to 5-day), RNAs were isolated from both embryonic and extraembryonic territories. For HH32, HH37, HH41, and HH45 samples (7-day, 10-day, 15-day, and 20-day), RNAs were isolated from the embryonic territory only. Multiple embryos (2–32) were used for the collection of samples younger than 3 days old, and single embryos were used for 3-day to 20-day samples. The extraembryonic samples were prepared from extraembryonic tissues (the amnion, chorion, allantois, and yolk sac) collected from HH32 and HH41 embryos. The HH41 extraembryonic sample had low sequencing reads and was not included in the PCoA. The fore- and hind-limb bud samples were collected from eggs purchased from Granja Gibert (Cambrils, Spain). The eggs were incubated to stage HH20. Fore- and hind-limb buds were dissected separately in cold PBS and RNA extracted with Trizol. Primary cell samples were purchased from Cell Applications (aortic SMCs: #CAC35405, strain unknown; hepatocytes: custom preparation, Cornish cross strain; MSCs: custom preparation, Rhode Island red strain). For electroporation of sgRNA and dCAS9-VP160 expressing constructs, fertilized eggs were grown to stage HH2–HH3 in ovo. Embryos were then isolated, electroporated with expression constructs (1 ug/ul final concentration for each construct), and cultured ex ovo in a New culture setting as previously described [16,58]. Electroporated embryos were monitored periodically for GFP activity (co-electroporated), and after reaching HH10, the embryos were fixed and processed for RNA in situ hybridization [16]. For expression validation of limb bud–related genes, RNA in situ hybridization was performed in whole-mount following standard procedures using digoxigenin-labeled antisense riboprobes. The probes used were PRRX1, VEGFD, LHX9, HOXA11, CRABP1, and HOXD10 kindly provided by A. Nieto, M. Davey, and J. Fallon or generated by PCR at MR lab.

### CAGE library preparation, mapping, CAGE peaks definition, and association to known genes

CAGE libraries were prepared following a standard protocol using 5 ug of total RNA per sample [59,60]. Libraries were then subjected to sequencing on the HeliScope single-molecule sequencer following the manufacturer directions [1]. The sequencing data were aligned to the chicken galGal5 genome assembly using delve (downloadable from fantom.gsc.riken.jp/software/), which is a hidden Markov model–based alignment method developed in house. Sequences that aligned uniquely to the reference genome with 85% identity and 99% accuracy were kept for all downstream analyses. CAGE TSSs were defined for each sample by extracting the first position of all mapped reads and counting their abundance, collapsing all the overlapping positions relative to the strand orientation. Sample details and total mapped tags are listed in S1 Table. Peaks (promoters) were defined by applying DPI method as described previously [3]. Promoters were then associated to the closest gene within 500 bp (both upstream and downstream of the gene TSS). Both Ensembl and RefSeq gene models were used in order to achieve the highest coverage. The avian CAGE dataset is available for download at DDBJ (DNA Data Bank of Japan) with the accession number DRA004812.

### Data processing

Expression normalization was calculated sample by sample as TPM. An additional normalization step was performed by R bioconductor edgeR package [61], normalization option “RLE” (relative log expression). Expression enrichment calculation for identification of sample/stage-specific promoters was calculated as the logarithm base 10 of the normalized expression over the median expression across all samples. Several functions in the edgeR package were also used for differential expression (DE) analysis between pairs of sample replicates, GO enrichment analysis for differentially expressed genes, and PCoA plots showing the development progression and the samples’ separations. Raw data for PCoA can be accessed at http://fantom.gsc.riken.jp/5/suppl/Lizio_et_al_2017/.

### Analysis of promoter features

CpG island boundaries were downloaded from UCSC genome browser and the overlaps with TSSs were calculated using bedtools “intersectBed” function. The SI and the GC percent calculation were based on the same procedure applied previously [33]. Promoters were extended up to 100 bp (± 50 bp) from the representative TSS position (the position with highest expression) before GC percentage and SI calculation and were subdivided into stage-, tissue- and non-specific in order to assess differences in promoter shape and GC content preference. Grouping into super clusters was achieved by applying the function “mergeBed” from the bedtools suite, by requiring a merging distance of 100 bp between TSSs. This distance ensures that single peak TSSs remain as such, while neighboring TSSs become part of a broader, multimodal promoter. In order to assess promoter size shifts, super clusters were defined for all robust TSSs expressed in 26 samples (the approximately 31,000 peaks) and also for each stage- and cell-type group separately (aortic, hepatic, mesenchymal, early devel, mid devel, mid-late devel, late devel, buds, extra-embryo). To generate super clusters for each separate group, only TSSs that expressed 1 TPM and above in at least 1 sample of the group were considered, to make them comparable to the entire set of robust TSSs.

### TFBS motif analysis

For TFBS motif analysis, (−300, +100) bp regions around the TSSs were extracted by bedtools 2.25. The extracted sequences were then scanned for local enrichment estimation using AME tool (MEME Suite 4.11.2) separately for each of the following subgroups of promoters: aortic, mesenchymal and hepatic primary cells, and early, middle, mid-late, and late embryo developmental tissues. Housekeeping TSSs were also tested as a separate group. The motifs that the TSSs were compared against were taken from JASPAR CORE database for vertebrates (2016). The top 50 enriched motifs (adjusted *p*-value ≤ 0.05) for embryo (all embryo development subgroups), primary cell (aortic, msc, and hepatocyte), and other tissue (buds, extraembryonic) groups were selected and compared to the housekeeping group in order to identify common motifs.

## Supporting information

S1 Fig CAGE TSSs annotated to known genes. A) Pie chart representing the proportion of peaks that are annotated by any of the gene models used (blue), and those that aren’t (yellow). B) Breakdown of the peak-gene associations (blue region in A) by gene model. C) Proportions of genes associated to a CAGE peak versus the non-associated. Although RefSeq gene set is much smaller than the Ensembl one, more RefSeq genes are correctly annotated, as shown by higher association to a CAGE peak. Numerical values for this plot can be found in supplementary file “S1 Data”.
(TIF)

S2 Fig Screenshot of Chicken-ZENBU view sections in the GAPDH gene locus. Detailed description of the view utilized in this study, with *GAPDH* locus as an example. 1: Terms search box. 2: Chromosome coordinates of the visualized region. 3: Chromosome band representation. 4: Entrez gene locus represented as a strand-oriented arrow (green: forward strand, purple: reverse strand). 5: Ensembl gene model track. 6: RefSeq gene model track. 7: Non-coding RNA genes tracks, minimized. 8 and 9: Other genes annotations (EST, repeats), minimized. 10: CAGE peaks tracks (permissive and robust), minimized. Genomic coordinates only are visualized when open. 11: Conserved elements tracks, minimized. Bird HCE: all highly conserved elements. ASHCE: avian specific highly conserved elements, after removing those that are also conserved in mammals. 12: CAGE TSS expression profiles for pooled samples, various grouping, minimized. 13: Typical CAGE signal profile at TSS. 14: Expression profiles of all the 16 embryo development samples (green: forward strand, purple: reverse strand). Split view of this will show expression profile in each sample. 15: Bar graph showing expression level in each sample. 16–19: Settings and visualization controls.
(TIF)

S3 Fig Screenshot of Chicken-ZENBU view of TSS peaks in the NANOG and GFAP gene loci. A) *NANOG*; B) *GFAP*. TSS peak positions and levels are indicated in the rectangular box (red-colored peak: reverse strand transcription). Peak levels are also shown as bar graph at the bottom. Samples are ordered in their temporal sequence (HH1 to HH45). High levels of *NANOG* expression at early developmental stages become rapidly downregulated. GFAP levels show a rapid increase after day 10.
(TIF)

S4 Fig Screenshot of Chicken-ZENBU view of TSS peaks in the CDYL gene locus. A) CAGE identifies two alternative promoters, indicated as TSS1 and TSS2 (red-colored peak: reverse strand transcription), not associated to any Ensembl gene model and with different expression patterns. B) Bar graphs showing that TSS1 exhibits stable expression throughout development while TSS2 has an early stages specific expression pattern. C) Graph visualizing the trend in expression at all 26 developmental stages (x-axis). Expression values are TPM displayed on a logarithmic scale. Numerical values for this plot can be found in supplementary file “S1 Data”.
(TIF)

S5 Fig Limb-enriched genes. A) Expression levels and TSS positions shown in Chicken-ZENBU screenshots of limbs specific (*PRRX1* and *VEGFD*), wing-enriched (*CRABP1* and *HOXD10*) and leg-enriched (*LHX9* and *HOXA11*) genes. B) Bar graph showing the absolute TPM expression of those genes. Numerical values for this plot can be found in supplementary file “S1 Data”. C-E) In situ hybridization expression analysis of enriched genes listed in A.
(TIF)

S6 Fig Top 30 enriched housekeeping genes. Visualization of expression for the top 30 enriched genes shown in Fig 4. Genes are sorted by max TPM expression (high to low). They are expressed at lower levels than *GAPDH* and *ACTB*, but are more stable and therefore more suitable as chicken housekeeping genes. Numerical values for this plot can be found in supplementary file “S1 Data”.
(TIF)

S7 Fig General features of chicken promoters. Shape, GC content and CpG island association of TSS peaks are analyzed. Peak shape is defined by shape index (SI) [33] and is categorized as either sharp (SI > -1) or broad (SI < -1). GC content is analyzed in a 100 bp region (+/- 50 bp from representative TSS position). A) Broad TSS peaks (SI < -1) are associated with a higher GC content. Red: associated with annotated genes. Green: not associated with annotated genes. B) A position weight matrix visualization for the 100 bp promoter regions of “sharp” and “broad” TSS peaks. Association with the CG-rich motif is seen for broad peaks. Association with the TATA box is seen for sharp peaks. C) Histogram showing the size of CpG-associated (red) non-CpG associated TSS peaks. CpG associated peaks tend to be longer. D) On average, promoter GC content is higher for non-specific TSSs than for stage- or tissue-specific TSS. E) SI distribution for promoters of different categories. Sharp peaks are more likely to be associated with stage- or tissue-specific TSSs than with non-specific TSSs. F) Percentage of CpG-associated TSSs (red) and non-CpG-associated TSSs (blue) in each sample. Total TSSs (>3TPM) in each sample is shown at the top. G) Density plot showing variation in super cluster sizes (x-axis) at different developmental groups. All: all samples combined. Super clusters tend to become larger at later stages, suggesting acquisition of new TSSs for a given promoter.
(TIF)

S8 Fig Chicken-ZENBU view of avian conserved elements (HCE and ASHCE). Regions around *MSX1* and *MSX2* genes are shown as examples, together with HCE and ASHCE regulatory elements in their vicinity. Color intensity reflects the level of conservation.
(TIF)

S9 Fig Venn diagram showing overlaps of the top 50, top 100 and all motifs for embryo, primary cell and other tissue groups against the housekeeping group. Top row: top 50; Middle row: top 100; Bottom row: all. Left column: developmental stages; Middle column: tissue specific cell types; Right column: limb buds and others.
(TIF)

S10 Fig Comparison of TFBS analysis of all TSSs with either sharp or broad TSS peaks. The top 50 enriched motifs for either sharp or broad TSS peaks (see the text for definition). Consensus sequences for the top 10 motifs are also shown. The sharp peaks are highly associated with TA rich motifs (left) and the broad peaks are highly associated with CG rich motifs (right).
(TIF)

S11 Fig Design of CRISPR-on sgRNAs for SNAI1, CDH1, MMP11 and DENND5B genes. A-D): Chicken-ZENBU views of these four genes. A: SNAI1; B: CDH1; C: MMP11; D: DENDD5B. A’-D’): Sequences of sgRNAs (red underline) located within 500 bp upstream of the TSS. Blue: protospacer adjacent motif (PAM). A’: SNAI1; B’: CDH1; C’: MMP11; D’:DENDD5B.
(TIF)

S12 Fig Induction of endogenous SNAI1, CDH1, MMP11 and DENDD5B expression mediated by CRISPR-on. Electroporated embryos were grown in New culture and sgRNA expressing territories were assessed by co-electroporated GFP signals. Embryos were processed for in situ hybridization (shown here). A, A’: SNAI1; B, B’: CDH1; C, C’: MMP11; D, D’: DENND5B. A-D: Whole embryo views. Black rectangles indicate areas with magnified views in A’-D’, with robust ectopic expression seen for all four genes. Endogenous expression of DENND5B is shown in panel E.
(TIF)

S1 Table CAGE sequencing data summary. The summary includes sample names, serial sample labels, category, number of CAGE sequence reads and number and percentage of TSS with expression above 3TPM.
(XLSX)

S2 Table Numbers of genes with single and multiple associated TSSs (>3TPM). About half of the genes (6274/12248) associated with mapped TSSs (>3TPM) have single TSS peak, and the other half of the genes have two or more TSS peaks. When taking into consideration of co-regulated multiple peaks (i.e., TSSs with similar expression profile across samples), about two thirds (8283/12248) are associated with a single expression cluster and the rest with one or more expression clusters.
(XLSX)

S3 Table Full set of stage and cell type specific TSS. This Supplementary table contains 11 separate tables. Expression enrichment scores are reported for all CAGE peaks (Sup-table3-DPIs_all). Lists of enriched peaks (score > 10) for each stage or cell type are also reported (Sup-table 3-early, Sup-table 3-mid, Sup-table 3-mid-late, sup-table 3 late, Sup-table 3-wing, Sup-table 3-leg, Sup-table 3-extraemb, Sup-table 3-hepatocytes, Sup-table 3-mesen and Sup-table 3-aortic).
(XLSX)

S4 Table Ubiquitously and highly active TSSs above 300TPM. This table lists all TSSs are highly active (>300TPM) in all samples. Many of them show high cross-sample variance.
(XLSX)

S5 Table Housekeeping genes with low variance and 10X fold expression. Full set of 1,254 housekeeping genes and corresponding log2(TPM) expression in all 26 samples. Statistical values (variance, standard deviation, fold change) are also reported.
(XLSX)

S6 Table Housekeeping genes conserved between human and chicken. This table cross-compares housekeeping genes between the human and the chicken. Left column: chicken unique genes. Middle column: genes share between these two species. Right column: human unique genes.
(XLSX)

S7 Table Gene ontology analysis of chicken housekeeping genes. This table lists enriched gene ontology terms associated with the chicken housekeeping genes. GO term column: Specific GO term with GO number. Ontology column: Category of ontology term. C: Cellular component; F: Molecular function; P: Biological process. GO terms are listed in the order of enrichment (high to low) for each of the three categories.
(XLSX)

S8 Table Enriched transcription factor binding site (TFBS) motifs in sub-groups of samples. This table contains 11 separate tables, listing TFBS motifs enriched in each of the 11 sub-groups. For each subgroup, AME, a local motif enrichment analysis, was performed using sequences located within -300/+100 base pairs of the TSSs. All enriched TFBS motifs are listed in this table.
(XLSX)

S9 Table Count tables for the Venn diagrams. This table has three separate tables, containing data for top 50, top 100 and all enriched motifs comparisons, respectively. Only the Venn diagram for top 50 motifs comparison is shown in Fig 5B.
(XLSX)

S1 Data This is a numerical values table for graphs shown in the main and supplementary figures. (XLSX)

## Abbreviations

AME: Analysis of Motif Enrichment
ASHCE: Avian-Specific Highly Conserved Element
CAGE: Cap Analysis of Gene Expression
CNEE: Conserved Non-Exonic Element
CRISPR: Clustered Regularly Interspaced Short Palindromic Repeat
DPI: decomposition-based peak identification
FANTOM: Functional Annotation of Mammalian Genome
GO: gene ontology
HCE: highly conserved element
HH: Hamburger and Hamilton
MSC: mesenchymal stem cell
PCoA: principal coordinates analysis
RACE: rapid amplification of cDNA end
RAMPAGE: RNA annotation and mapping of promoters for analysis of gene expression
sgRNA: single guide RNA
SI: shape index
SMC: smooth muscle cell
SMRT: single-molecule real-time
TFBS: transcription factor binding site
TPM: tag per million
TSS: transcription start site
