# Supplementary figures and images for: Systematic analysis of transcription start sites in avian development
Source: PLoS Biol. 2017 Sep 5;15(9):e2002887. doi: 10.1371/journal.pbio.2002887 (PMC5600399; doi:10.1371/journal.pbio.2002887)

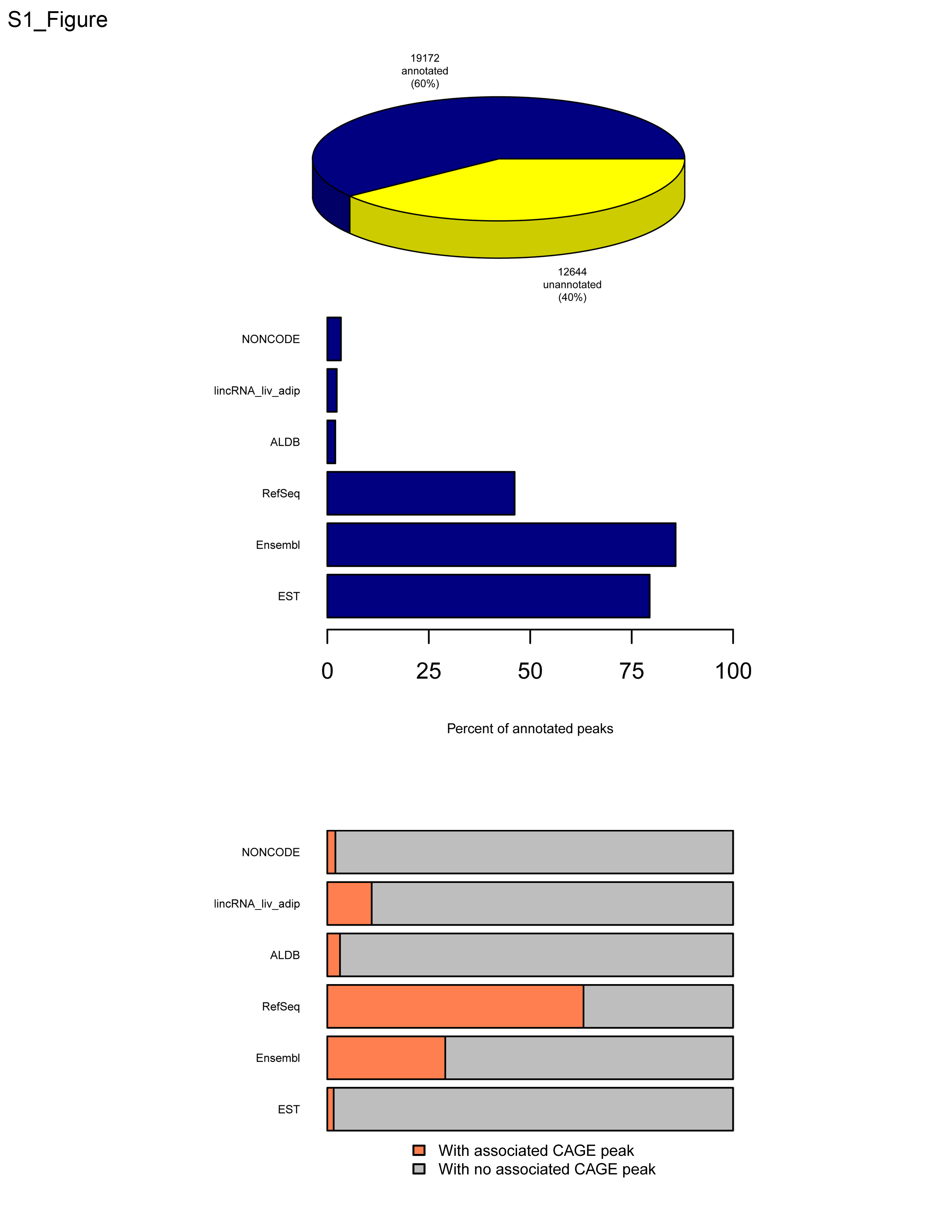

Supplement: S1 Fig — A) Pie chart representing the proportion of peaks that are annotated by any of the gene models used (blue), and those that aren’t (yellow). B) Breakdown of the peak-gene associations (blue region in A) by gene model. C) Proportions of genes associated to a CAGE peak versus the non-associated. Although RefSeq gene set is much smaller than the Ensembl one, more RefSeq genes are correctly annotated, as shown by higher association to a CAGE peak. Numerical values for this plot can be found in supplementary file “S1 Data”. (TIF) [file pbio.2002887.s001.tif]

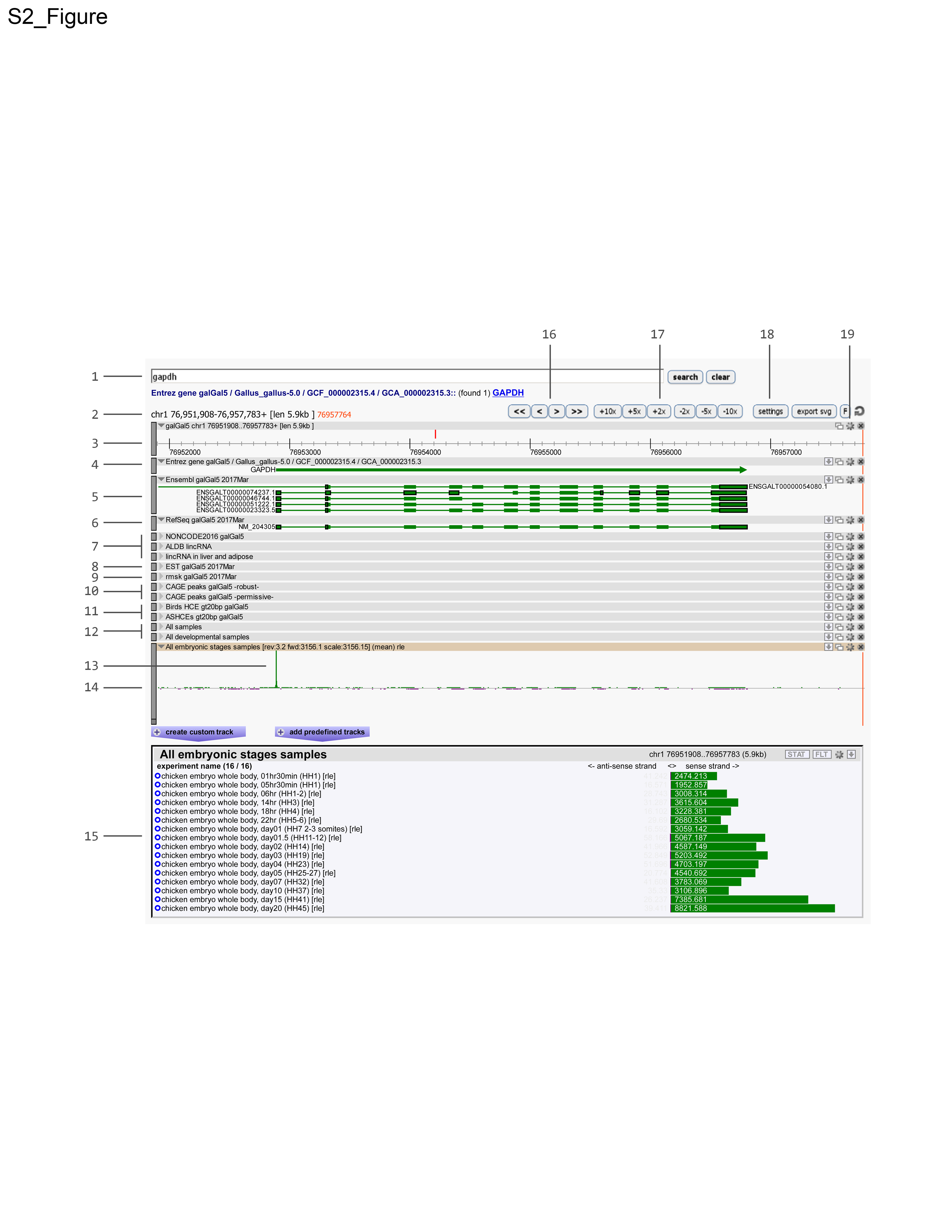

Supplement: S2 Fig — Detailed description of the view utilized in this study, with GAPDH locus as an example. 1: Terms search box. 2: Chromosome coordinates of the visualized region. 3: Chromosome band representation. 4: Entrez gene locus represented as a strand-oriented arrow (green: forward strand, purple: reverse strand). 5: Ensembl gene model track. 6: RefSeq gene model track. 7: Non-coding RNA genes tracks, minimized. 8 and 9: Other genes annotations (EST, repeats), minimized. 10: CAGE peaks tracks (permissive and robust), minimized. Genomic coordinates only are visualized when open. 11: Conserved elements tracks, minimized. Bird HCE: all highly conserved elements. ASHCE: avian specific highly conserved elements, after removing those that are also conserved in mammals. 12: CAGE TSS expression profiles for pooled samples, various grouping, minimized. 13: Typical CAGE signal profile at TSS. 14: Expression profiles of all the 16 embryo development samples (green: forward strand, purple: reverse strand). Split view of this will show expression profile in each sample. 15: Bar graph showing expression level in each sample. 16–19: Settings and visualization controls. (TIF) [file pbio.2002887.s002.tif]

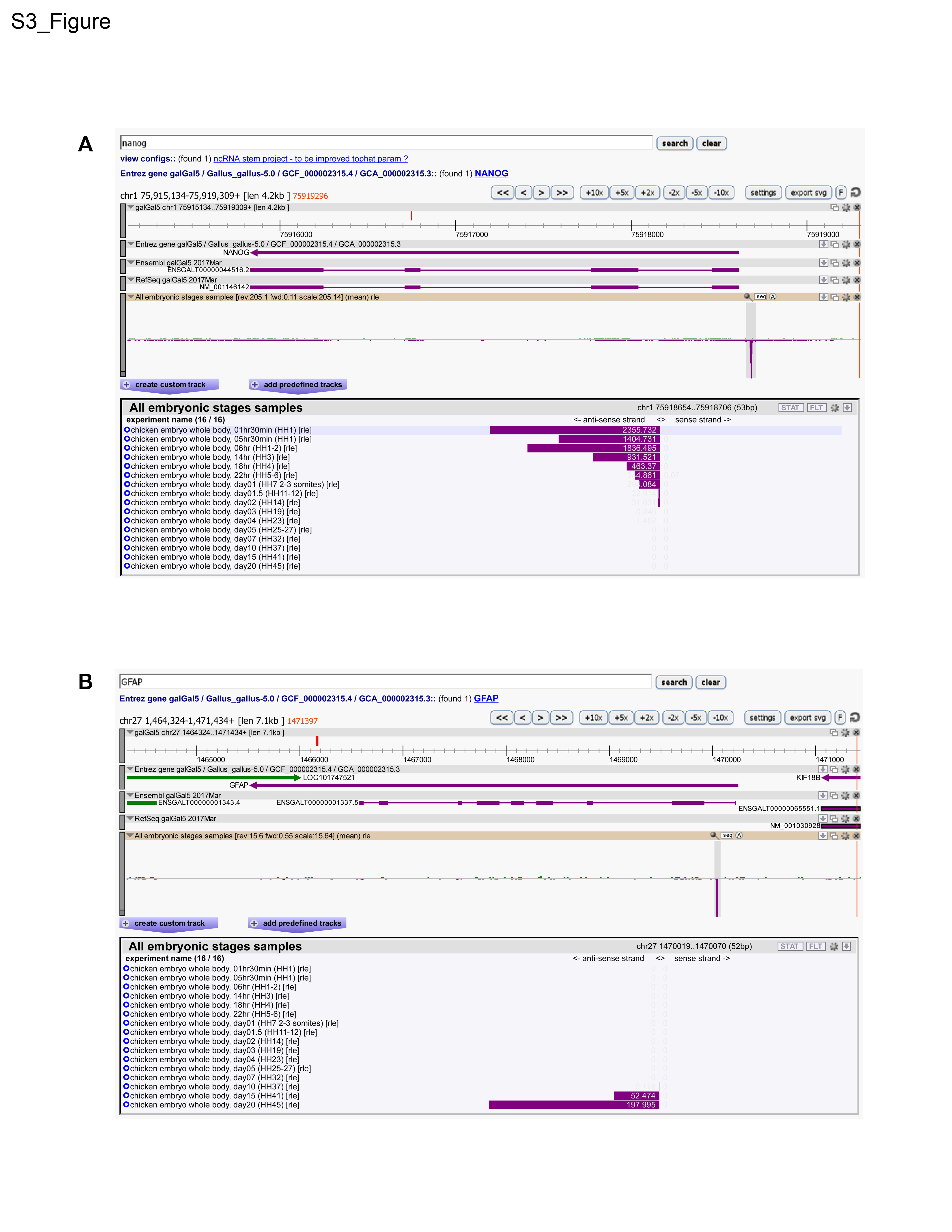

Supplement: S3 Fig — A) NANOG; B) GFAP. TSS peak positions and levels are indicated in the rectangular box (red-colored peak: reverse strand transcription). Peak levels are also shown as bar graph at the bottom. Samples are ordered in their temporal sequence (HH1 to HH45). High levels of NANOG expression at early developmental stages become rapidly downregulated. GFAP levels show a rapid increase after day 10. (TIF) [file pbio.2002887.s003.tif]

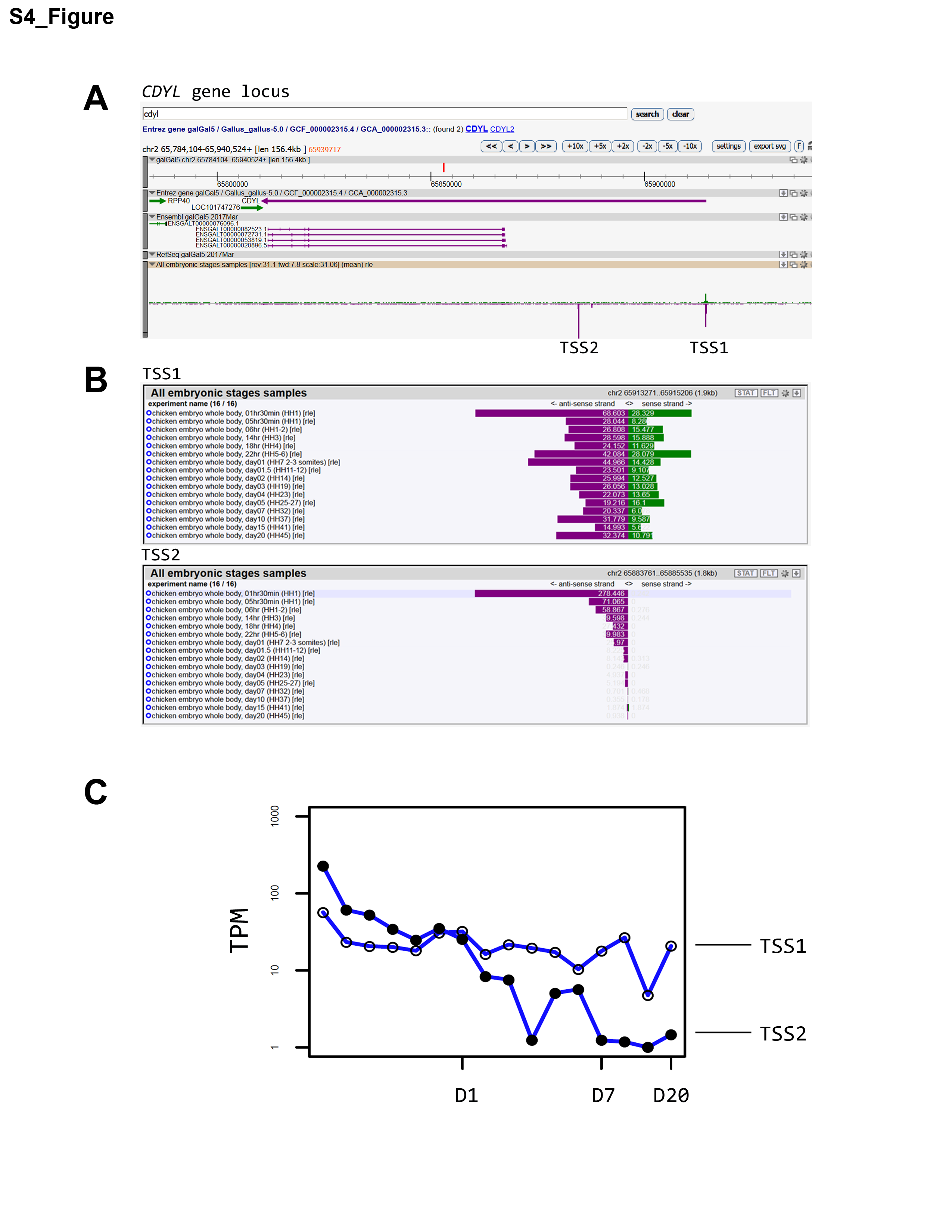

Supplement: S4 Fig — A) CAGE identifies two alternative promoters, indicated as TSS1 and TSS2 (red-colored peak: reverse strand transcription), not associated to any Ensembl gene model and with different expression patterns. B) Bar graphs showing that TSS1 exhibits stable expression throughout development while TSS2 has an early stages specific expression pattern. C) Graph visualizing the trend in expression at all 26 developmental stages (x-axis). Expression values are TPM displayed on a logarithmic scale. Numerical values for this plot can be found in supplementary file “S1 Data”. (TIF) [file pbio.2002887.s004.tif]

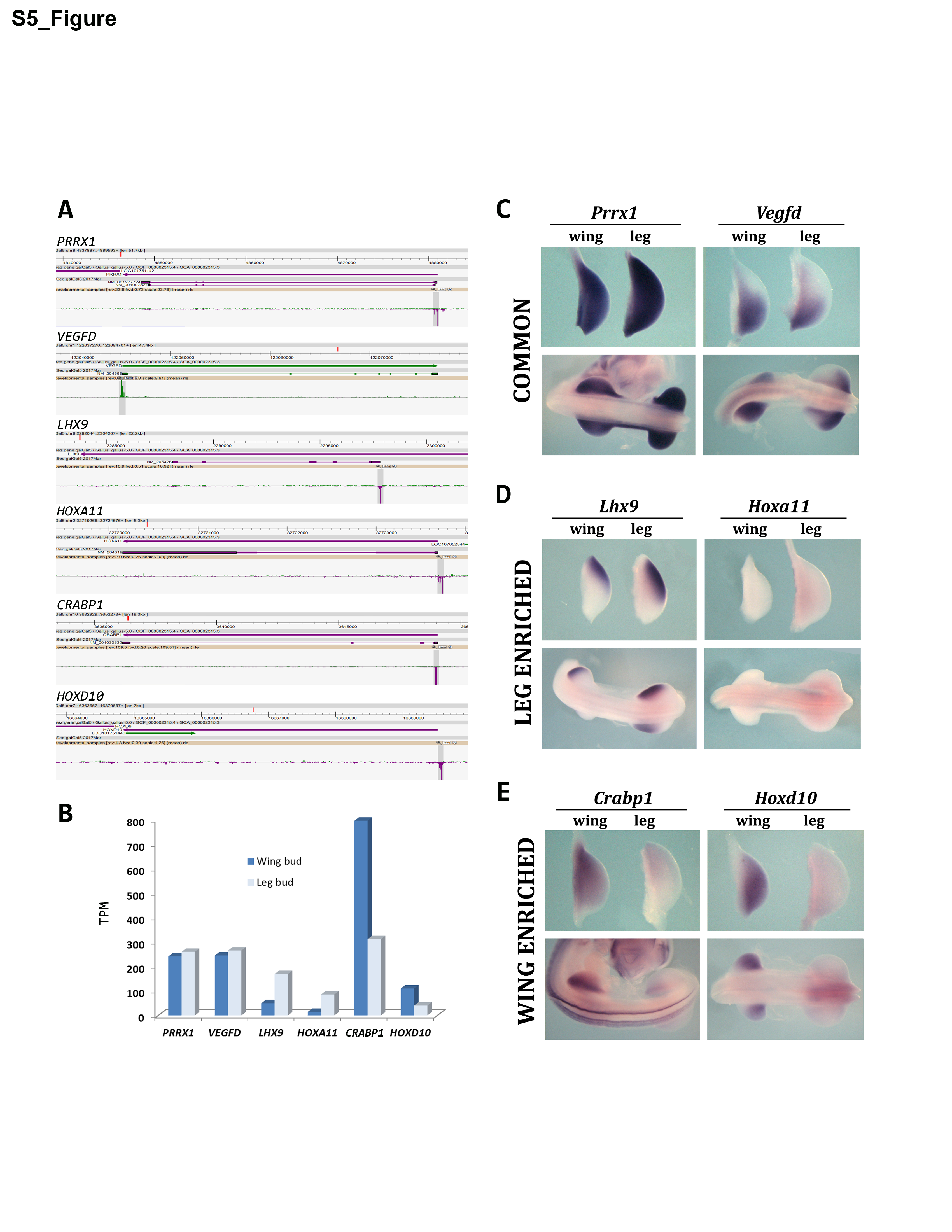

Supplement: S5 Fig — A) Expression levels and TSS positions shown in Chicken-ZENBU screenshots of limbs specific (PRRX1 and VEGFD), wing-enriched (CRABP1 and HOXD10) and leg-enriched (LHX9 and HOXA11) genes. B) Bar graph showing the absolute TPM expression of those genes. Numerical values for this plot can be found in supplementary file “S1 Data”. C-E) In situ hybridization expression analysis of enriched genes listed in A. (TIF) [file pbio.2002887.s005.tif]

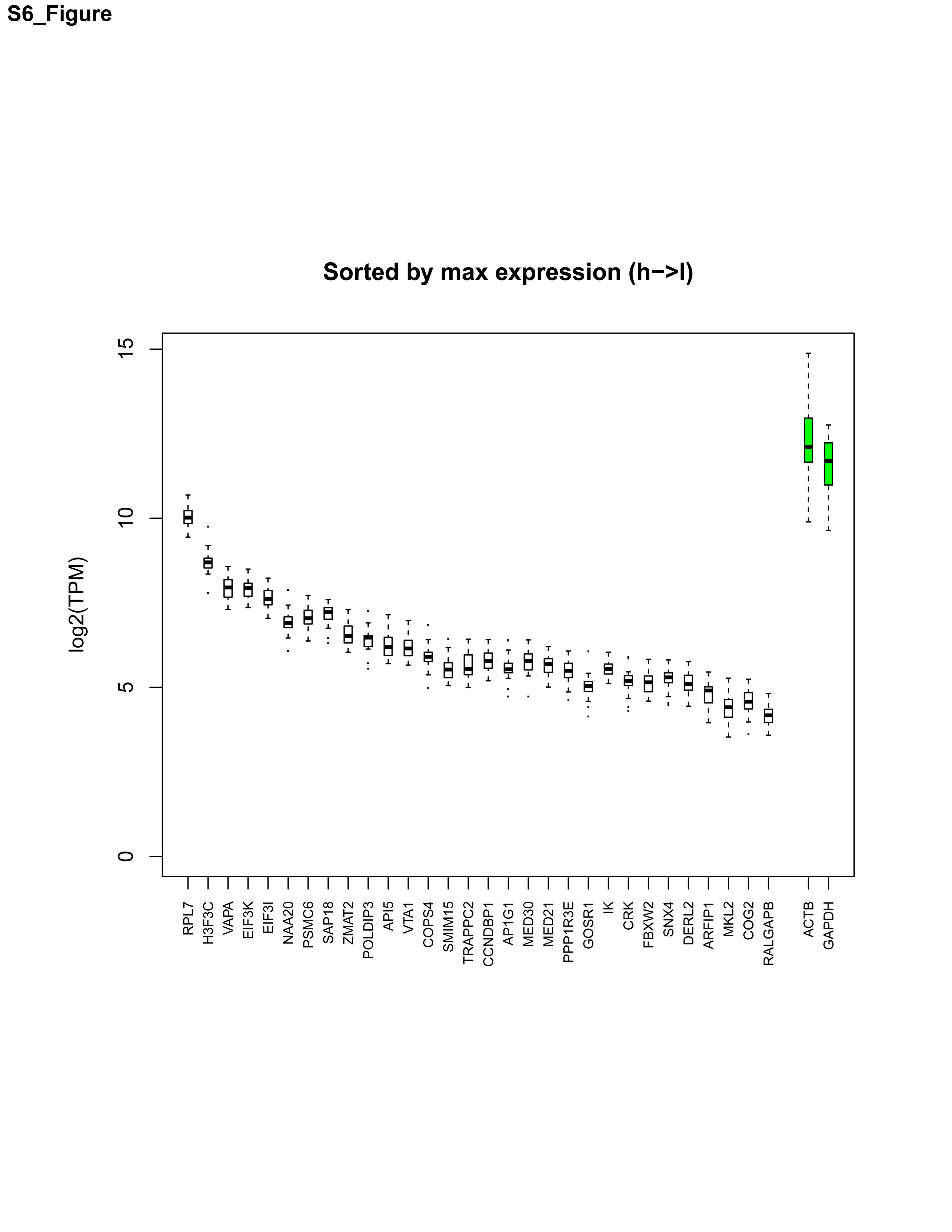

Supplement: S6 Fig — Visualization of expression for the top 30 enriched genes shown in Fig 4. Genes are sorted by max TPM expression (high to low). They are expressed at lower levels than GAPDH and ACTB, but are more stable and therefore more suitable as chicken housekeeping genes. Numerical values for this plot can be found in supplementary file “S1 Data”. (TIF) [file pbio.2002887.s006.tif]

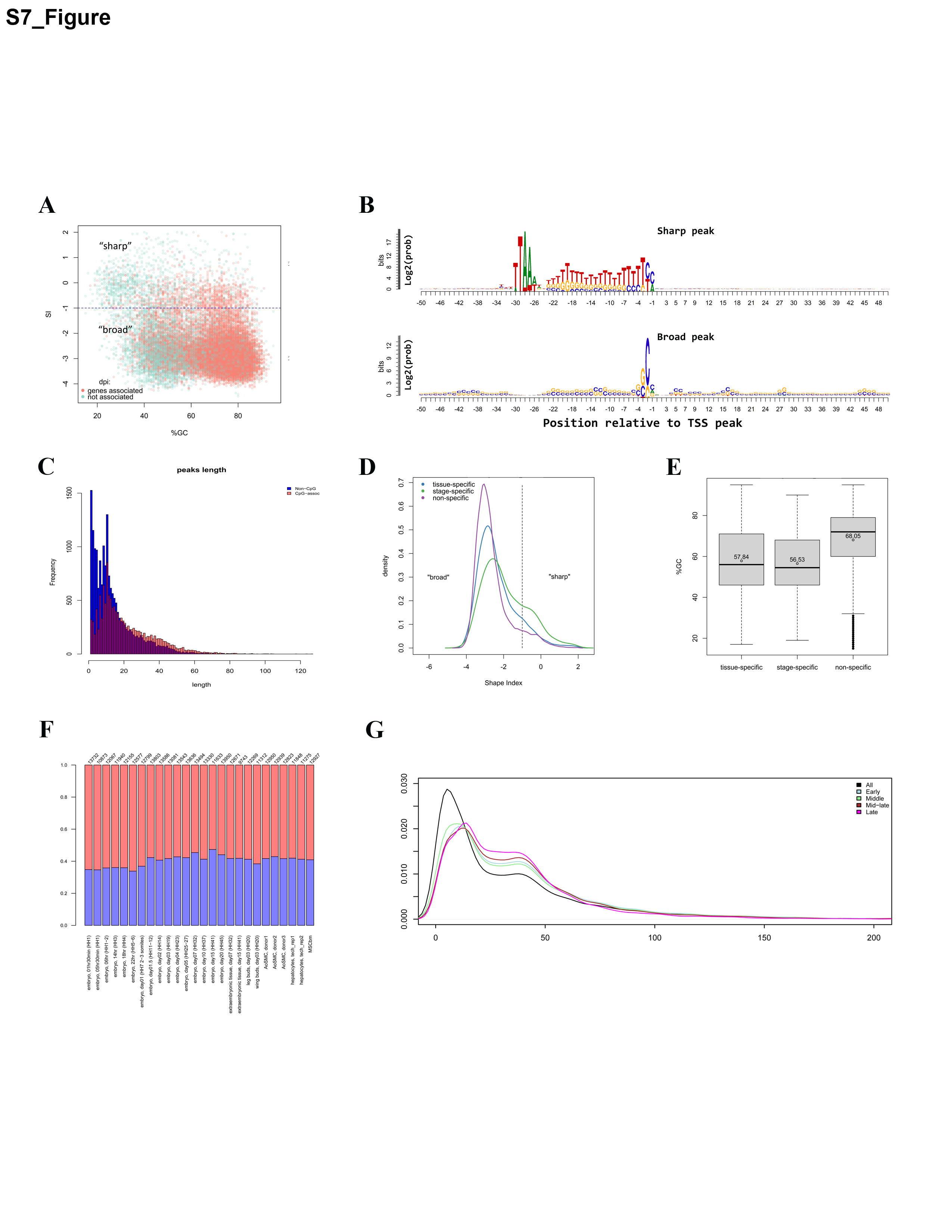

Supplement: S7 Fig — Shape, GC content and CpG island association of TSS peaks are analyzed. Peak shape is defined by shape index (SI) [33] and is categorized as either sharp (SI > -1) or broad (SI < -1). GC content is analyzed in a 100 bp region (+/- 50 bp from representative TSS position). A) Broad TSS peaks (SI < -1) are associated with a higher GC content. Red: associated with annotated genes. Green: not associated with annotated genes. B) A position weight matrix visualization for the 100 bp promoter regions of “sharp” and “broad” TSS peaks. Association with the CG-rich motif is seen for broad peaks. Association with the TATA box is seen for sharp peaks. C) Histogram showing the size of CpG-associated (red) non-CpG associated TSS peaks. CpG associated peaks tend to be longer. D) On average, promoter GC content is higher for non-specific TSSs than for stage- or tissue-specific TSS. E) SI distribution for promoters of different categories. Sharp peaks are more likely to be associated with stage- or tissue-specific TSSs than with non-specific TSSs. F) Percentage of CpG-associated TSSs (red) and non-CpG-associated TSSs (blue) in each sample. Total TSSs (>3TPM) in each sample is shown at the top. G) Density plot showing variation in super cluster sizes (x-axis) at different developmental groups. All: all samples combined. Super clusters tend to become larger at later stages, suggesting acquisition of new TSSs for a given promoter. (TIF) [file pbio.2002887.s007.tif]

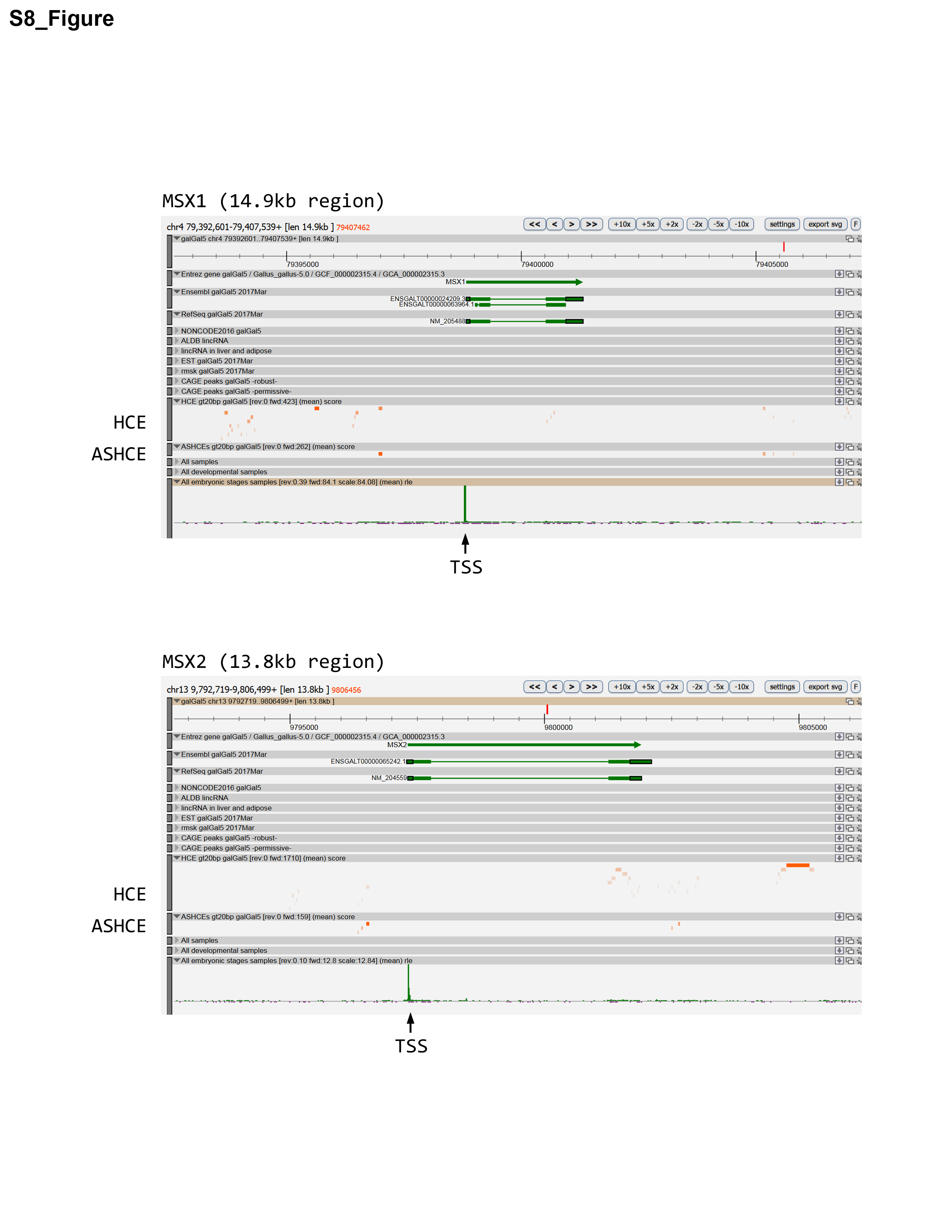

Supplement: S8 Fig — Regions around MSX1 and MSX2 genes are shown as examples, together with HCE and ASHCE regulatory elements in their vicinity. Color intensity reflects the level of conservation. (TIF) [file pbio.2002887.s008.tif]

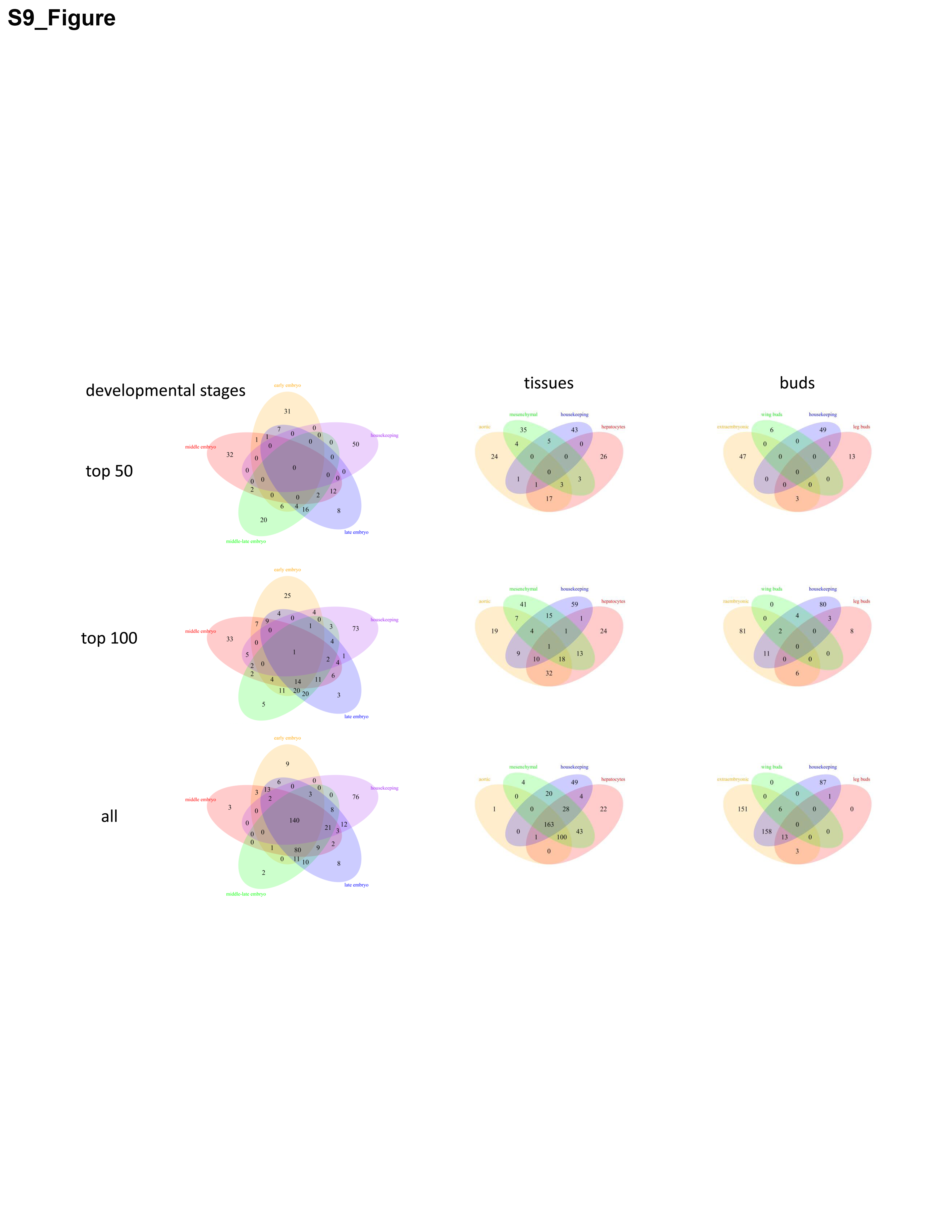

Supplement: S9 Fig — Top row: top 50; Middle row: top 100; Bottom row: all. Left column: developmental stages; Middle column: tissue specific cell types; Right column: limb buds and others. (TIF) [file pbio.2002887.s009.tif]

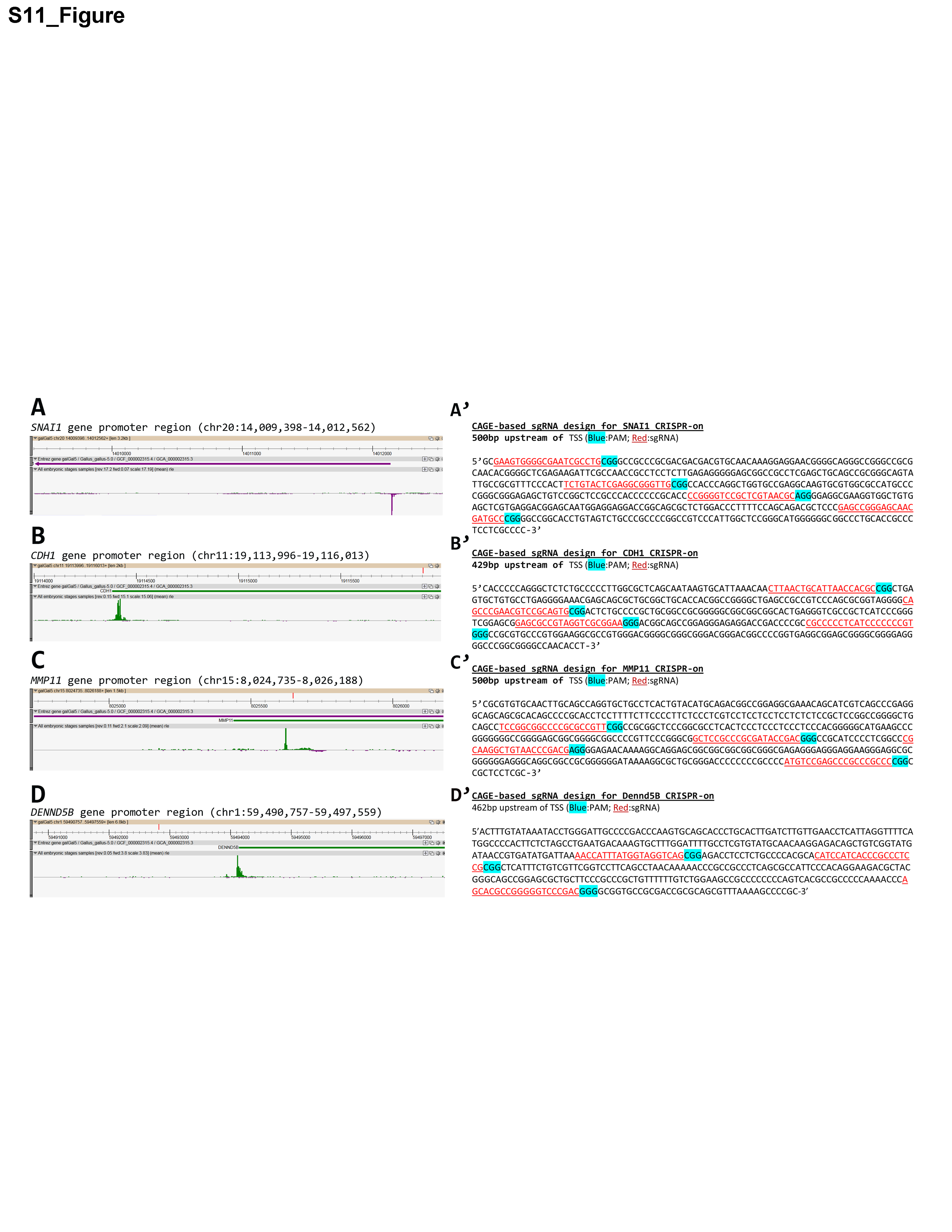

Supplement: S11 Fig — A-D): Chicken-ZENBU views of these four genes. A: SNAI1; B: CDH1; C: MMP11; D: DENDD5B. A’-D’): Sequences of sgRNAs (red underline) located within 500 bp upstream of the TSS. Blue: protospacer adjacent motif (PAM). A’: SNAI1; B’: CDH1; C’: MMP11; D’:DENDD5B. (TIF) [file pbio.2002887.s011.tif]

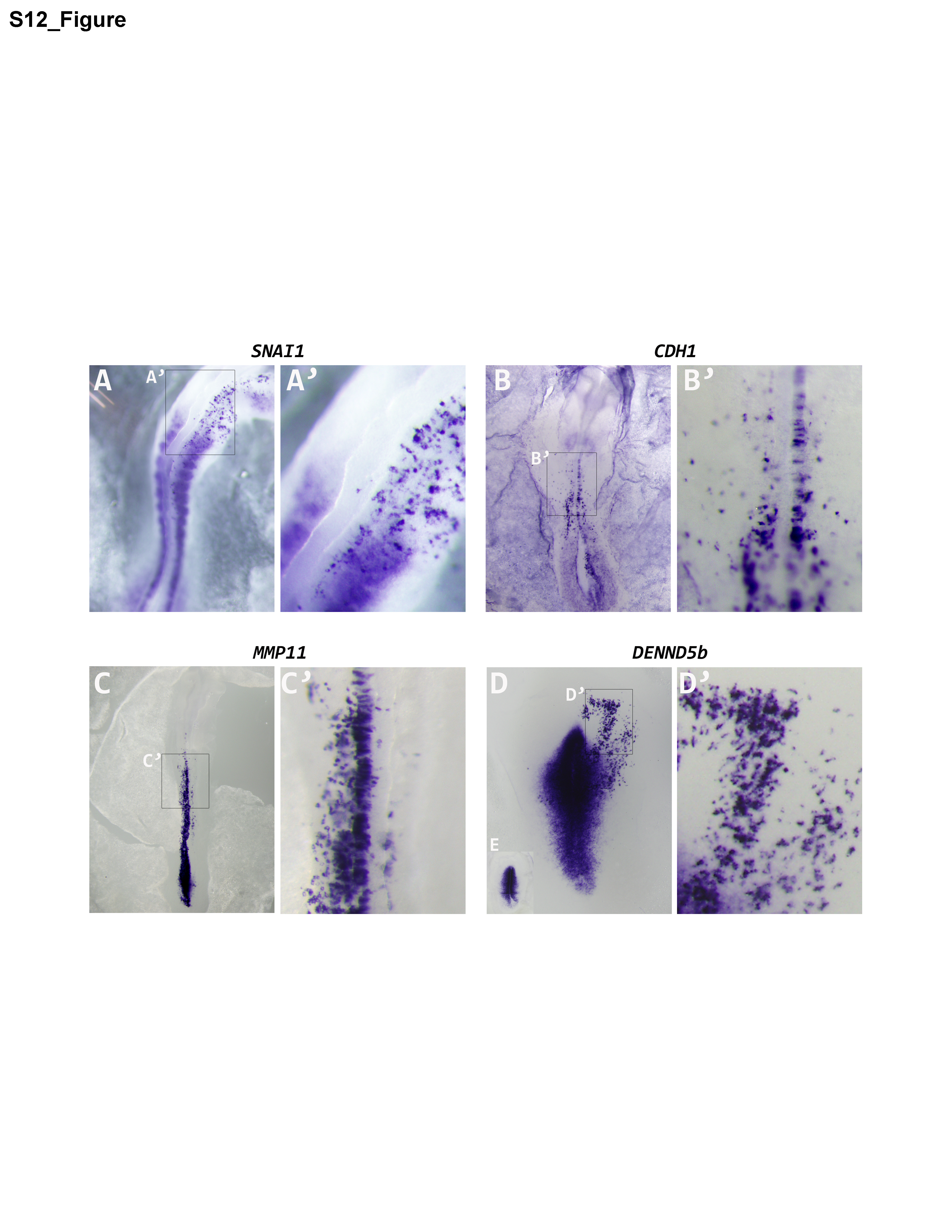

Supplement: S12 Fig — Electroporated embryos were grown in New culture and sgRNA expressing territories were assessed by co-electroporated GFP signals. Embryos were processed for in situ hybridization (shown here). A, A’: SNAI1; B, B’: CDH1; C, C’: MMP11; D, D’: DENND5B. A-D: Whole embryo views. Black rectangles indicate areas with magnified views in A’-D’, with robust ectopic expression seen for all four genes. Endogenous expression of DENND5B is shown in panel E. (TIF) [file pbio.2002887.s012.tif]
